# *OsJAZ11* regulates spikelet and seed development in rice

**DOI:** 10.1002/pld3.401

**Published:** 2022-05-10

**Authors:** Poonam Mehra, Bipin K. Pandey, Lokesh Verma, Ankita Prusty, Ajit Pal Singh, Shivam Sharma, Naveen Malik, Malcolm J. Bennett, Swarup K. Parida, Jitender Giri, Akhilesh K. Tyagi

**Affiliations:** ^1^ Department of Plant Molecular Biology University of Delhi South Campus New Delhi India; ^2^ National Institute of Plant Genome Research New Delhi India; ^3^ Plant and Crop Sciences, School of Biosciences University of Nottingham Sutton Bonington UK

**Keywords:** JASMONATE ZIM‐DOMAIN, Jasmonic acid, seed weight, seed width

## Abstract

Seed size is one of the major determinants of seed weight and eventually, crop yield. As the global population is increasing beyond the capacity of current food production, enhancing seed size is a key target for crop breeders. Despite the identification of several genes and QTLs, current understanding about the molecular regulation of seed size/weight remains fragmentary. In the present study, we report novel role of a jasmonic acid (JA) signaling repressor, *OsJAZ11* controlling rice seed width and weight. Transgenic rice lines overexpressing *OsJAZ11* exhibited up to a 14% increase in seed width and ~30% increase in seed weight compared to wild type (WT). Constitutive expression of *OsJAZ11* dramatically influenced spikelet morphogenesis leading to extra glume‐like structures, open hull, and abnormal numbers of floral organs. Furthermore, overexpression lines accumulated higher JA levels in spikelets and developing seeds. Expression studies uncovered altered expression of JA biosynthesis/signaling and MADS box genes in overexpression lines compared to WT. Yeast two‐hybrid and pull‐down assays revealed that OsJAZ11 interacts with OsMADS29 and OsMADS68. Remarkably, expression of *OsGW7*, a key negative regulator of grain size, was significantly reduced in overexpression lines. We propose that *OsJAZ11* participates in the regulation of seed size and spikelet development by coordinating the expression of JA‐related, *OsGW7* and MADS genes.

## 1 INTRODUCTION

Rice serves as a primary source of energy for >3.5 billion people worldwide (Seck et al., 2012). To feed the global population, it is essential to create new rice varieties with higher yield potential. Seed weight, number of seeds, and number of panicles are considered major yield components in rice (Song et al., 2007). Of these, seed weight is usually determined by shape parameters such as seed length, width, and thickness. To date, many important QTLs regulating grain length and/or width have been identified. For instance, *GS3*, *GL3.1*, *GW6a*, *TGW3*, and *TGW6* (Fan et al., 2006; Ishimaru et al., 2013; Qi et al., 2012; Song et al., 2015; Ying et al., 2018) have been characterized for grain length. *GW2*, *GW5*, *GS5*, and *GW8/OsSPL16* regulate grain width (Li et al., 2011; Song et al., 2007; Wang et al., 2012; Weng et al., 2008). Furthermore, *GW7/GL7* and *GS2/GL2* influence both grain length and width (Che et al., 2016; Hu et al., 2015; Wang et al., 2015; Zhang et al., 2013). However, little is known about the molecular mechanisms by which these genes determine final seed size and weight.

Jasmonic acid (JA) plays important roles in plants during responses to several biotic and abiotic stresses (reviewed in Wasternack & Hause, 2013; Trang Nguyen et al., 2019; Ali & Baek, 2020; Wang et al., 2020). Linolenic acid is the precursor of JA biosynthesis which is finally metabolized into the bioactive isoleucine conjugate, JA‐Ile (Yuan & Zhang, 2015). JA signaling is activated by the interaction between JA‐Ile and COI1 receptor (CORONATINE INSENSITIVE1), which leads to proteasomal degradation of a family of JAZ (JASMONATE ZIM‐DOMAIN) transcriptional repressor proteins (Chini et al., 2007; Yan et al., 2009). Degradation of JAZ repressors releases a set of transcription factors including MYC2 which activates the expression of JA‐responsive genes (Ali & Baek, 2020). JA biosynthesis and/or signaling regulate development of leaves, roots, pollen, stamen, trichome, tuber, xylem, and hypocotyl (reviewed in Huang et al., 2017; Jang et al., 2019). In rice, JA biosynthesis and signaling have been associated with spikelet morphogenesis (Cai et al., 2014; Hori et al., 2014; You et al., 2019). JA biosynthetic mutant, *eg1* (*extra glume 1*), showed abnormal spikelet morphology. *EG1* encodes for a plastid‐targeted class I lipase of the Phospholipase A1 family which is essential to produce JA precursors. In addition, another *extra‐glume* mutant (*eg2‐1D*) exhibited altered floral identity and defects in floral meristem determination (Cai et al., 2014). Map‐based cloning revealed that *EG2* encodes for a JAZ repressor, OsJAZ1. A detailed study uncovered that the spikelet defects in *eg2‐1D* are due to the suppressed action of the *OsMYC2* regulated E‐class gene, *OsMADS1*. Despite our increasing understanding of JA involvement in spikelet morphogenesis, the roles of this signal in the regulation of rice seed traits remain largely unknown.

JAZ repressors consist of a conserved TIFY or ZIM domain and a C‐terminal Jas domain. The conserved TIFY domain homo‐ or hetero‐dimerizes with JAZ proteins. The Jas motif is critical for binding of JAZ repressors with COI1 as well as downstream target MYC2 (Staswick, 2008). Both ZIM and Jas domain are required for JAZ‐mediated transcriptional repression of JA responses (Pauwels et al., 2010; Pauwels & Goossens, 2011). The rice genome encodes 15 JAZ repressors (Singh et al., 2015; Ye et al., 2009). Growing evidence reveals roles for JAZ repressors in plant defense, development, and abiotic stress tolerance (Cai et al., 2014; Hori et al., 2014; Pandey et al., 2021; Seo et al., 2011; Singh et al., 2020; Wu et al., 2015; Yamada et al., 2012). In the present study, we characterized a rice JAZ repressor, *OsJAZ11*, which shows high expression in different panicle and seed developmental stages. We report that *OsJAZ11* acts as a regulator of seed width and weight traits in rice. Our study also reveals novel JA‐dependent molecular mechanisms that regulate rice grain width and weight.

## 2 MATERIALS AND METHODS

### 2.1 Plant growth conditions and phenotyping

WT and transgenics rice, generated earlier (Pandey et al., 2021), were grown in soil pots in containment greenhouse conditions at 28°C, ~70% relative humidity, and 12/12 h (light/dark) photoperiod. Seed length and width traits were estimated in mature seeds using WinSEEDLE Pro (Regent Instruments Inc., Canada). Yield parameters such as 100 seed weight, yield per panicle, and yield per plant were measured manually using digital weighing balance. Number of filled and unfilled grains were measured manually. Pollen viability was analyzed by crushing anthers into I_2_‐KI solution (3:1) as described earlier (Ranjan et al., 2017). Viable and non‐viable pollen were counted under light microscope. Panicles and seed images were captured using Nikon‐5200 DSLR camera. Statistical analysis was performed using Student's *t* test.

### 2.2 Generation of *OsJAZ11* rice transgenics

Overexpression (OE), RNAi (Ri), and translational reporter lines of *OsJAZ11* were generated previously (Pandey et al., 2021). Briefly, for overexpression of *OsJAZ11* under *ZmUbi1* promoter, full length cDNA was cloned in pANIC6B overexpression vector. For generating RNAi lines, 350 bp region of *OsJAZ11* was cloned into silencing vector, pANIC8B. For generating translational reporters of *OsJAZ11*, protein coding region of *OsJAZ11* and truncated version of *OsJAZ11* (*OsJAZ11* ORF with 57 a.a. deleted at C‐terminal region) were amplified and fused in‐frame with GUS (*β‐glucuronidase*) reporter in pCAMBIA1301. For generating transcriptional reporters of *OsJAZ11*, 1.5 kb promoter region of *OsJAZ11* was cloned upstream of *GUS* in pMDC163 vector. All constructs were transformed into PB1 (*Oryza sativa* L.) by *Agrobacterium*‐mediated transformation as described previously (Mehra et al., 2017). Positive transformants were selected on hygromycin. Expression of *OsJAZ11* was also analyzed in spikelets by RT‐qPCR (Quantitative Real‐Time PCR). Experiments were finally performed in T3 homozygous lines. All primers used in cloning are listed in Table S1.

### 2.3 JA estimation

JA levels were measured in spikelets (P4 panicle stage) and seeds (S2 stage) using the method described previously (Lin et al., 2019; Pandey et al., 2021). Samples of 100 mg were crushed in liquid nitrogen and suspended in 1 ml of 1X PBS, pH 7.4. Supernatant fraction was separated by centrifuging samples at 3,000 rpm for 20 min. Thereafter, JA levels were measured using quantitative sandwich ELISA (enzyme‐linked immunosorbent assay) kit (MyBioSource, San Diego, USA) as per manufacturer's protocol. All estimations were made using six independent biological replicates.

### 2.4 Quantitative real‐time PCR

RT‐qPCR was performed using QuantStudio 3 Real‐Time PCR system (Thermo Scientific, USA) as described earlier (Pandey et al., 2021; Singh et al., 2015). *Ubiquitin5* was used as internal reference. Relative expression levels were calculated by 2^‐Δ(ΔCT)^ method. Primer sequences used for RT‐qPCR are listed in Table S1.

### 2.5 GUS histochemical and fluorometric assays

Rice transgenics expressing *pOsJAZ11:GUS* were raised in soil pots as described above. Samples were collected from six different stages of panicle development (determined by panicle length) in rice: P1 (.5–3 cm), P2 (3–5 cm), P3 (5–10 cm), P4 (10–15 cm), P5 (15–22 cm), and P6 (22–30 cm). Samples were also harvested from five different stages of seed development in rice depending on days after pollination (dap): S1 (0–2 dap), S2 (3–4 dap), S3 (5–10 dap), S4 (11–20 dap), and S5 (21–29 dap). All samples were subjected to histochemical GUS staining as described earlier (Mehra et al., 2019; Pandey et al., 2021). Images were captured using Zeiss stereo zoom microscope. For fluorometric MUG estimation, samples from *OsJAZ11‐GUS* and *OsJAZ11ΔC‐GUS* transgenics were frozen in liquid nitrogen, and MUG assays were carried out as described previously (Mehra et al., 2019, Pandey et al., 2021).

### 2.6 Immunoblotting with anti‐GUS antibody

A total of 150 mg of leaf sample was crushed in liquid nitrogen and incubated with extraction buffer (200 mM Tris‐Cl pH 8.0, 100 mM NaCl, 400 mM Sucrose, 10 mM EDTA, 100 mM PMSF, 0.05% Tween‐20, 28.6 mM β‐mercaptoethanol, 1X Protease Inhibitor Cocktail). Proteins were allowed to extract for 30 min at 4°C with gentle shaking. Samples were centrifuged at 13,000 rpm, 4°C, and supernatant was collected. Protein quantity was estimated with Bradford using BSA standards. A total of 40 μg of protein was electrophoresed on 12% SDS‐PAGE. Thereafter, proteins were blotted on to a polyvinylidene difluoride membrane (Hybond‐PVDF, Amersham, UK) according to the manufacturer's protocol. Membrane was blocked with 5% (w/v) skimmed milk in PBST buffer for 2 h at room temperature. Membrane was thereafter incubated with rabbit anti‐β‐glucuronidase (Invitrogen) (1:1,000) for 2 h followed by three gentle washing with PBST for 5, 10, and 5 min. Subsequently, membranes were incubated with secondary antibody (horseradish peroxidase‐labeled goat anti‐rabbit IgG) (Sigma‐Aldrich, USA) in 1:10,000 dilution. Membrane was washed twice with PBST for 5 and 10 min. Thereafter, GUS signals were detected using ECL Prime western blotting detection kit (GE Healthcare Biosciences, UK) as per manufacturer's protocol. Equal amount of protein loading was assured with Ponceau‐S staining.

### 2.7 Yeast two‐hybrid (Y2H) assay

Protein–protein interactions assays in yeast cells were performed using Matchmaker Gold Yeast two‐hybrid system (Clonetech, USA) as described previously (Pandey et al., 2021). Briefly, coding sequences of target proteins were cloned into bait vector (BD, pGBKT7) or prey vector (AD, pGADT7). AD and BD clones were co‐transformed in Y2H Gold yeast strain. Positive interactions were screened on ‐HLT medium (SD/‐Leu/‐Trp/‐His), ‐AHLT medium (SD/‐Leu/‐Trp/‐His/‐Ade), and ‐AHLT medium supplemented with X‐alpha‐gal and Aureobasidin A (SD/‐Leu/‐Trp/‐His/‐Ade/X‐Gal/Aureobasidin A). All primers used for Y2H assays are listed in Table S1.

### 2.8 In vitro pull‐down assay

Protein coding region of *OsJAZ11* was cloned into expression vector, pGEX‐4T‐1 to overexpress recombinant OsJAZ11‐GST. Coding region of OsJAZ1, OsMADS68, and OsMADS29 was cloned in expression vector, pET28a, to overexpress recombinant OsJAZ1‐6XHIS, OsMADS68‐6XHIS, and OsMADS29‐6XHIS, respectively. All vector constructs were independently transformed and induced into *Escherichia coli* strain, BL21(DE3)pLysS, as described earlier (Pandey et al., 2017). Proteins from bacterial cells were extracted and purified as described previously (Mehra & Giri, 2016; Pandey et al., 2021). In vitro pull‐down assay was performed as described earlier (Pandey et al., 2021).

## 3 RESULTS

### 3.1 *OsJAZ11* is highly expressed in developing rice panicles and seeds

To determine the expression patterns of *OsJAZ11*, *pOsJAZ11*:*GUS* transgenic lines were generated in rice and subjected to histochemical β‐glucuronidase (GUS) assays. GUS signals were detected in all panicle development stages and early stages of seed development (Figure S1). Strong expression was observed in P3 and P4 stages of panicle development. GUS signals weakened as seeds progressed toward maturity. Among the various reproductive organs, strong expression was detected in anthers, lemma, palea, and awns. The observed expression patterns in different tissues were consistent with microarray data retrieved from RiceXPro (Sato et al., 2010) (Figure S2).

### 3.2 OsJAZ11 protein accumulates in panicle development stages

OsJAZ11 undergoes JA‐dependent proteasome‐mediated degradation (Pandey et al., 2021). Therefore, it is imperative to study their protein dynamics for understanding their functional roles in different physiological and developmental processes. In order to analyze OsJAZ11 accumulation in panicle and seed development stages, we employed two different translational reporters (*OsJAZ11‐GUS* and *OsJAZ11ΔC‐GUS*) of *OsJAZ11* in rice (Pandey et al., 2021). These translational reporters overexpress GUS fusions of full‐length OsJAZ11 (OsJAZ11‐GUS) or truncated version of OsJAZ11, that is, OsJAZ11ΔC (OsJAZ11ΔC‐GUS) under *CaMV35S* promoter in rice (Figure S3). Notably, OsJAZ11ΔC lacks 57 amino acid residues at C‐terminal region which also includes the Jas motif. Jas motif is critical for interaction of JAZ proteins with COI1, an F‐box protein that recruits SCF‐type E3 ubiquitin ligase (SCF^COI1^) and mediates degradation of OsJAZ11 via 26S proteasome (Pandey et al., 2021). Hence, deletion of Jas motif would inhibit degradation of OsJAZ11 resulting in constitutive repression of JA signaling.

**FIGURE 1:**
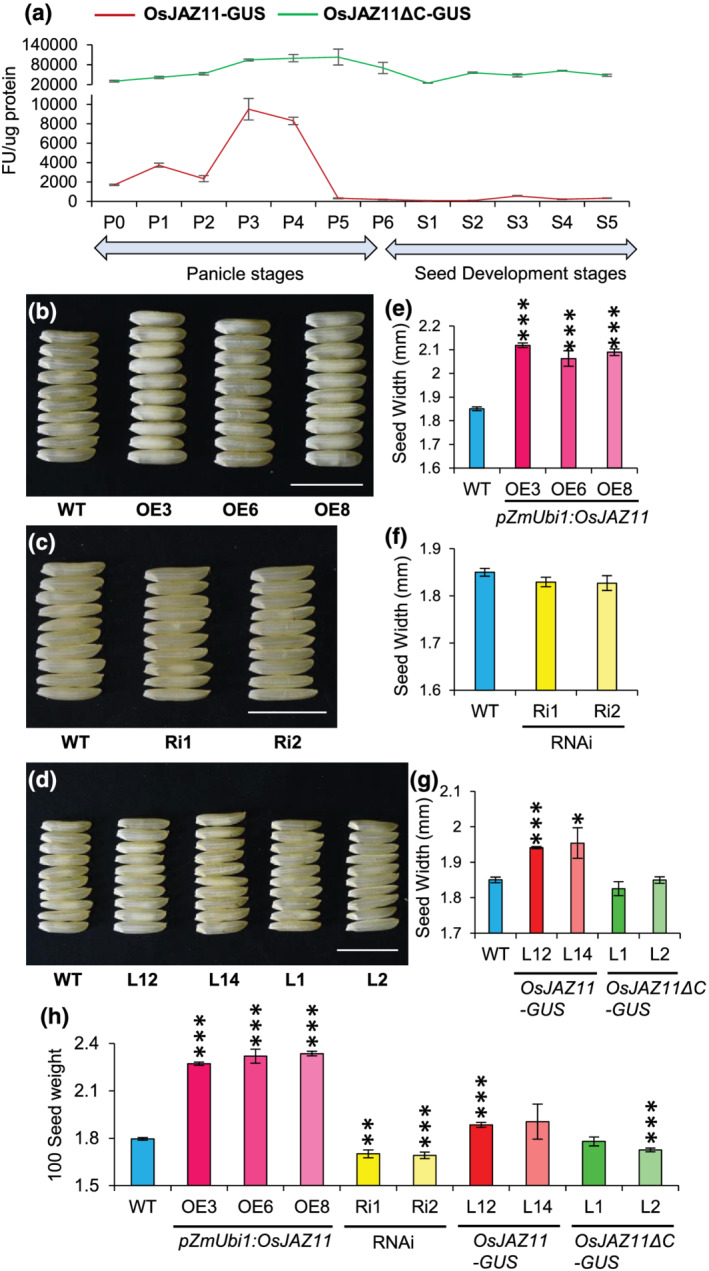
*OsJAZ11* regulates seed width and weight. (a) Quantitation of GUS activity by MUG assay in *pCAMV35S*:*OsJAZ11‐GUS* (L14) and *pCAMV35S*:*OsJAZ11ΔC‐GUS* (L2)*.* GUS activity was determined in different panicle (P0 to P6) and seed (S1 to S5) developmental stages (*n* = 6). P0 refers to panicle length up to 0.5 mm or shoot apical meristem. (b–d) Comparison of seed phenotype of WT and *OsJAZ11* overexpression lines (*pZmUbi1*:*OsJAZ11*; OE3/6/8), RNAi lines (Ri1/2), *OsJAZ11‐GUS* lines (L12/L14), and *OsJAZ11ΔC‐GUS* lines (L1/2). Bar = 1 cm. (e–g) Quantitation of seed width in WT and *OsJAZ11* transgenics. Each bar represents average of 100 seeds. (h) One‐hundred seed weight (in grams) of WT and *OsJAZ11* transgenics (*n* = 4). The same WT control has been used in b–d and e–g for comparison between WT and transgenics. Error bar represents standard error. Significant differences between WT and transgenics were determined using Student's *t* test. *, **, and *** represent *p* values ≤.05, .01, and .001, respectively

Immunoblotting assays also revealed higher accumulation of OsJAZ11 in panicles of *OsJAZ11ΔC‐GUS* transgenics as compared to *OsJAZ11‐GUS* lines (Figure S3d). We next quantitated GUS signals in panicle and seed development stages of both translational reporters. In *OsJAZ11‐GUS* lines, higher levels of OsJAZ11 protein accumulated in panicle development stages than seed development stages. Very high accumulation of OsJAZ11 was observed in P3 and P4 panicle stages (Figure 1a). After P4 panicle stage, accumulation of OsJAZ11 steeply declines and becomes more or less stable till seed maturation. These results indicate roles of *OsJAZ11* in early reproductive development stages of rice. On the other hand, as expected, OsJAZ11ΔC protein showed very high and stable accumulation throughout all reproductive stages (Figure 1a), explaining resistance to degradation of OsJAZ11ΔC by proteasomal machinery.

### 3.3 Overexpression of *OsJAZ11* enhances seed width and weight in rice

To study functional roles of *OsJAZ11* in regulating seed traits, we employed previously generated overexpression (OE) and silencing (Ri) lines of *OsJAZ11* in rice (Pandey et al., 2021). Overexpression lines showed significantly higher expression of *OsJAZ11* in spikelet tissues than WT, whereas silencing lines showed only marginal decrease in *OsJAZ11* transcripts in spikelet tissues (Figure S4). Seed traits of both transgenics were compared to WT after maturation stage. Notably, overexpression of *OsJAZ11* significantly increased seed width (11–14%) and weight (26–30%) as compared to WT (Figure 1b,e,h). Though Ri lines showed only slight (~ 1%) reduction in seed width in comparison to WT, substantial decrease in 100 seed weight (~5.8%) was also observed in Ri lines (Figure 1c,f,h). Contrary to seed width, seed length was significantly less (2.8–3.5%) in OE lines than WT, whereas we did not find any significant difference in seed length between WT and Ri lines (Figure S5). These results suggest *OsJAZ11* positively regulates rice grain weight by increasing seed width. Interestingly, translational reporter lines, *CaMV35S:OsJAZ11‐GUS*, also displayed increased seed width (~5%) and seed weight (5–6%) as compared to WT (Figure 1d,g,h). This further confirmed the importance of *OsJAZ11* in rice seed width regulation. Significant changes in seed weight and seed width were also consistent with observations in T2 generation of *OsJAZ11* transgenics (Figure S6).

### 3.4 *OsJAZ11* overexpression reduces grain filling and yield

As *OsJAZ11* improves grain size and weight, we further examined other yield parameters in *OsJAZ11* transgenics. At maturity, OE lines produced smaller panicles than WT (Figures S7a and S8a). We observed 11–18% decrease in panicle length in OE lines in comparison to WT. Similar to OE lines, *CaMV35S:OsJAZ11‐GUS* and *CaMV35S:OsJAZ11ΔC‐GUS* also displayed significant decrease in panicle length (Figures S7 and S8c). On the other hand, Ri lines exhibited no alterations in panicle length as compared to WT (Figures S7b and S8b). Apart from reduced panicle length, the average number of seeds per panicle also reduced to 68–76% in OE lines compared to WT (Figure S9). Notably, only 36–52% florets showed grain filling in OE lines compared to WT (Figure S10). Reduced panicle length and seed setting collectively led to reduced number of seeds per panicle in OE lines than WT. Due to this, OE lines also exhibited 2.4‐ to 4.0‐fold decrease in yield per panicle and 2.4‐ to 3.5‐fold decrease in yield per plant as compared to WT (Figure S11). These results reveal substantial yield penalty in *OsJAZ11* OE lines as compared to WT. Moreover, due to smaller panicle length, *OsJAZ11‐GUS* and *OsJAZ11ΔC‐GUS* reporters also showed significant decrease in number of seeds per panicle, yield per panicle, and yield per plant (Figures S9 and S11). However, no defects in seed setting were observed in any of the translational reporters (Figure S10). Contrary to OE lines, Ri lines depicted no significant differences in seed setting or yield as compared to WT (Figures S10 and S11). Insignificant differences in Ri lines may also be attributed to marginal silencing of *OsJAZ11* in Ri lines.

### 3.5 Overexpression of *OsJAZ11* causes pleiotropic defects in spikelet morphology

**FIGURE 2:**
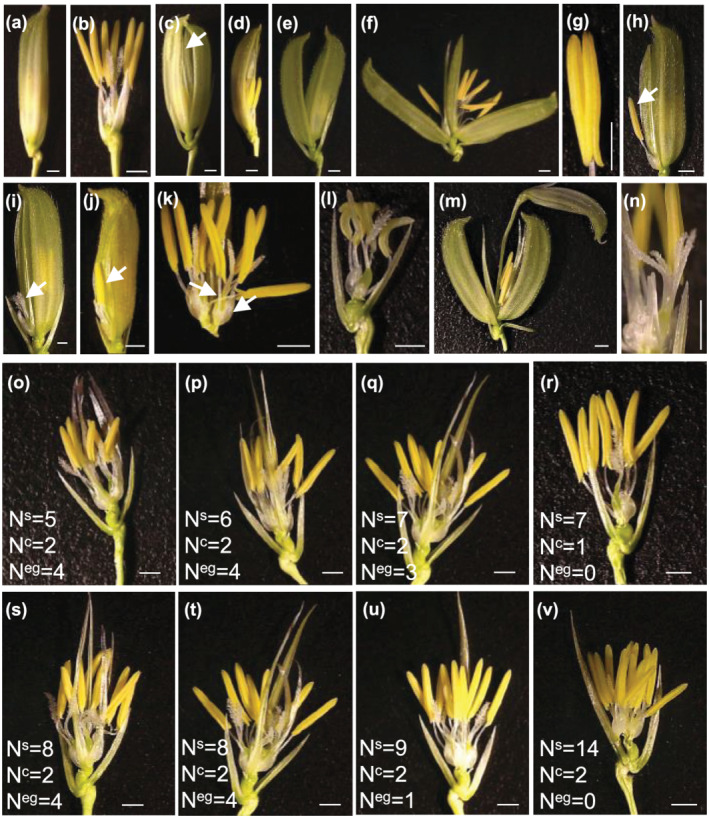
Phenotypes of spikelet in *OsJAZ11* overexpression transgenics. (a, b) Normal spikelet morphology of WT. (c) Abnormal spikelets of *OsJAZ11* OE line showing extra glume‐like structure (indicated by arrow), (d) missing palea, (e, f) extra lemma, (g) fused anthers, (h) misplaced stamen protruding out of spikelet, (i) misplaced carpel protruding out of spikelet, (j) stamen and carpel outside spikelet, (k) supernumerary stamens and carpels, (l) curled anthers, (m) spikelet within spikelet, and (n) multiple stigma. (o–v) *OsJAZ11* OE spikelets with abnormal number of stamens (N^s^), carpels (N^c^) and extra glume like structures (N^eg^). Bar = 1 mm

Wild‐type spikelet of rice generally has two pairs of leafy structures (rudimentary glumes and sterile lemmas) and one fertile floret. The organization of a fertile floret consists of four whorls. First whorl consists of a lemma and a palea, second whorl is comprised of two lodicules, third whorl has six stamens, and fourth whorl possesses one pistil with two stigmas. Spikelet phenotyping of *OsJAZ11* OE lines revealed multiple defects in spikelet morphogenesis (Figure 2). However, no defects in floral organs were observed in any of the RNAi and translational reporter lines (Figure S12). On an average, 36–49% of the spikelets in OE lines exhibited one or more type of spikelet defects (Table S2). Notably, most of the defected spikelets (31–56%) showed open hull phenotype. Open hull spikelets in OE lines could be easily spotted before heading of the panicles (Figure S13). Most of the spikelets formed extra glume‐like structures between the sterile lemma and whorl 1 (Figure 2). The number of extra glumes ranged from 0 to 4 (Table S3). Apart from extra glume‐like structure, we also observed supernumerary or fewer stamens (3–14), multiple carpels (1–2), or multiple lodicules (2–4) (Figure 2; Table S3). Many of the spikelets also showed missing palea, extra lemma/palea (hull), fused anthers, curled anthers, multiple stigma, and ectopic florets (Figure 2). Interestingly, few of the spikelets showed misplaced floral organs with stamens and/or carpels protruding outside the floret (Figure 2). These results suggest that *OsJAZ11* is involved in pathways that regulate spikelet development and floral identity determination.

We next examined the effect of defective spikelet morphology on pollen viability of OE lines. Therefore, we classified all OE spikelets in to three major classes (normal‐hull, open‐hull, and missing‐hull spikelets) and analyzed their pollen viability (Figure S14). Normal‐hull spikelets of *OsJAZ11* OE lines showed pollen viability similar to WT, though many of the normal‐hull spikelets also displayed abnormal number of floral organs (e.g., stamens, carpels, or glumes). Pollen viability was substantially compromised in spikelets with open‐full or missing‐hull phenotypes suggesting desiccation of microspores in exposed anthers prior to dehiscence. Decreased pollen viability may also be the key factor behind reduced seed set and yield in OE lines as compared to WT. On the other hand, Ri lines and translational reporters showed normal pollen viability owing to their normal spikelet morphology (Figure S15).

### 3.6 Overexpression of *OsJAZ11* alters JA signaling and biosynthesis in rice

**FIGURE 3:**
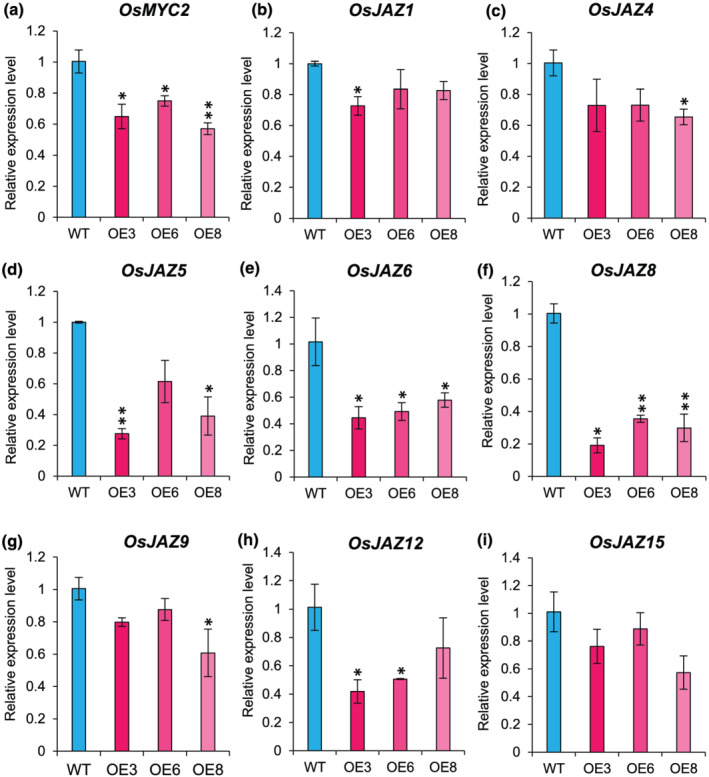
Overexpression of *OsJAZ11* suppresses JA signaling in rice. (a) Relative expression levels of *OsMYC2*, (b) *OsJAZ1*, (c) *OsJAZ4*, (d) *OsJAZ5*, (e) *OsJAZ6*, (f) *OsJAZ8*, (g) *OsJAZ9*, (h) *OsJAZ12*, and (i) *OsJAZ15* in WT and *OsJAZ11* OE lines. Expression levels were analyzed in spikelets at P4 stage of development (*n* = 3). Error bar represents standard error. Significant differences between WT and transgenics were determined using Student's *t* test. * and ** represent *p* values ≤.05 and .01, respectively

JAZ repressors are integral components of JA signaling pathway; therefore, their abundance may disrupt the JA signaling and/or JA biosynthesis. To elucidate the impact of *OsJAZ11* overexpression on JA signaling, we first analyzed the expression levels of key JA signaling genes (*OsCOI1*, *OsMYC2*, and several JAZ repressors) in *OsJAZ11* OE lines. This expression analysis revealed significant downregulation of *OsMYC2*, *OsJAZ5*, *OsJAZ6*, *OsJAZ8*, and *OsJAZ12* (Figure 3) suggesting suppression of JA signaling in OE lines. Notably, OsJAZ11 also oligomerizes with many of the rice JAZ repressors (OsJAZ1, OsJAZ4, OsJAZ5, OsJAZ6, OsJAZ8, OsJAZ9, OsJAZ12, and OsJAZ15) (Figures S16 and S17). However, no self‐dimerization was observed in OsJAZ11 (Figure S17). It is interesting to note here that *osjaz1* mutant also displays extra glume phenotype similar to *OsJAZ11* OE lines (Cai et al., 2014). We further confirmed the interaction between OsJAZ11 and OsJAZ1 by in vitro pull‐down assay (Figure S17b). Interaction of OsJAZ11 with other JAZ repressors may also affect JA response pathways in OE lines.

**FIGURE 4:**
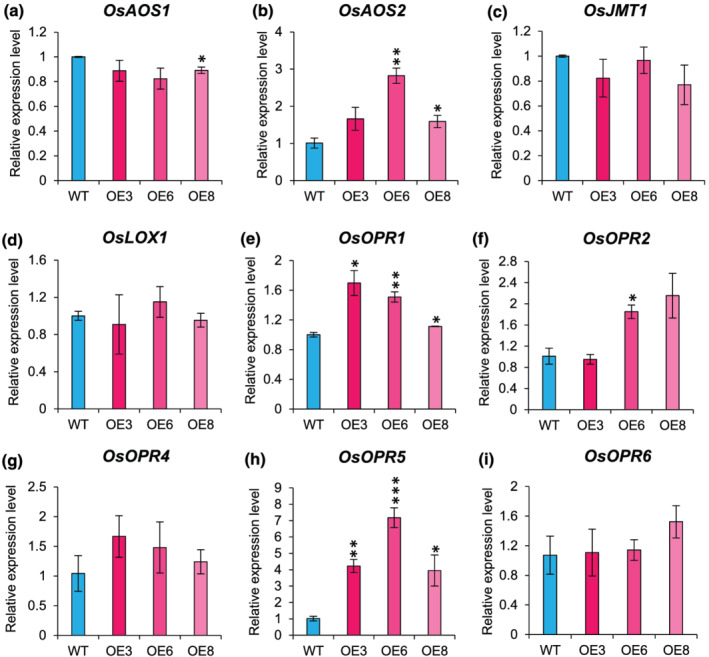
Overexpression of *OsJA11* increased expression of JA biosynthesis genes. Expression profile of (a) *OsAOS1*, (b) *OsAOS2*, (c) *OsJMT1*, (d) *OsLOX1*, (e) *OsOPR1*, (f) *OsOPR2*, (g) *OsOPR4*, (h) *OsOPR5*, and (i) *OsOPR6* in WT and *OsJAZ11* OE lines. Expression levels were analyzed in spikelets at P4 stage of development (*n* = 3). Error bar represents standard error. Significant differences between WT and transgenics were determined using Student's *t* test. *, **, and *** represent *p* values ≤.05, .01, and .001, respectively

**FIGURE 5:**
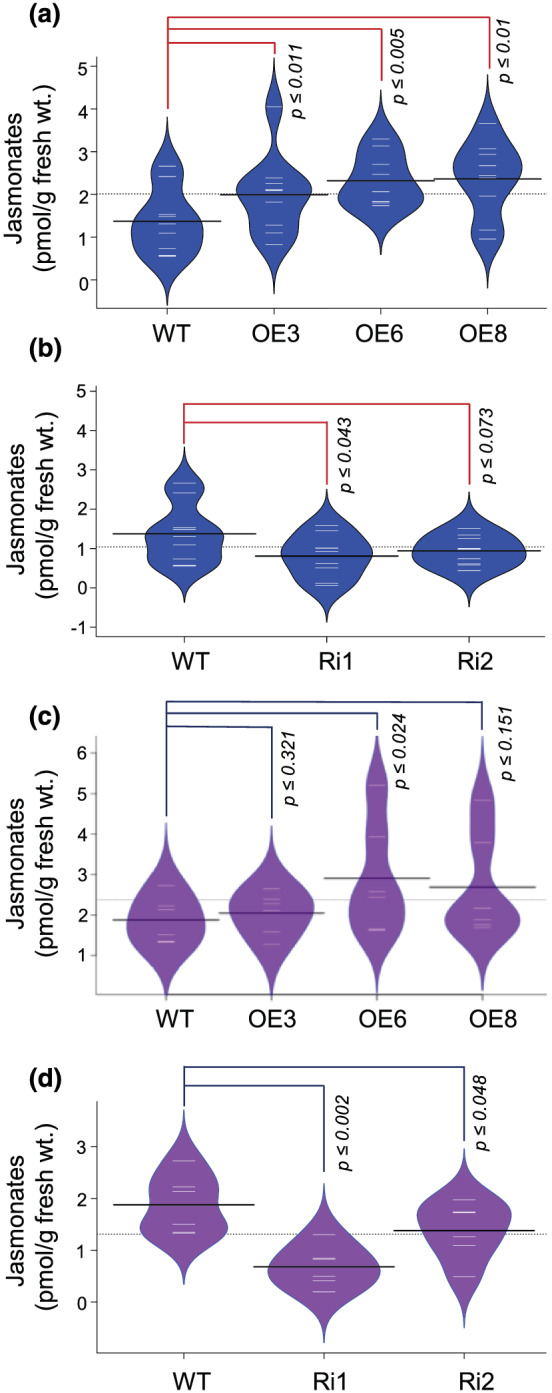
*OsJAZ11* overexpressing lines showed increased JA biosynthesis. (a, b) JA content/g fresh weight of P4 spikelets of WT, *OsJAZ11* OE, and Ri lines (*n* = 9). (c, d) JA content/g fresh weight of S2 seeds of WT, *OsJAZ11* OE, and Ri lines (*n* = 6). Each white line in a bean represents data from single plant. Black line in each bean represents average for each line. *p* values were determined using Student's *t* test

Second, we examined the expression levels of key JA biosynthetic genes (*OsAOS1/2*, *OsLOX1*, *OsJMT1*, and *OsOPR1/2/4/5/6*) in OE lines (Figure 4). As compared to WT, *OsAOS2*, *OsOPR1*, *OsOPR2*, and *OsOPR5* were upregulated in OE lines (Figure 4). Higher expression of JA biosynthetic genes indicates increased biosynthesis of JA in OE lines than WT. Therefore, we next estimated JA levels in WT and transgenic plants in P4 panicle stage. Our investigations reveal significantly higher JA biosynthesis in OE lines as compared to WT (Figure 5a). Marginal decrease in JA levels were also detected in Ri lines compared to WT (Figure 5b). Additionally, we also measured JA levels in developing seeds (S2 stage). In this case also, we observed higher JA levels in OE lines and lower JA levels in Ri lines as compared to WT (Figure 5c,d). These findings indicate that overexpression of *OsJAZ11* led to suppression of JA signaling in rice. These events in a feed‐back loop probably switched on a JA biosynthesis pathway in OE lines.

### 3.7 *OsJAZ11* regulates expression of MADS transcription factors

MADS‐domain transcription factors (TFs) play essential roles in determination of floral identity and development of floral meristem (Callens et al., 2018; Chongloi et al., 2019). Since *OsJAZ11* OE lines displayed multiple spikelet defects, we investigated the possible roles of MADS‐box genes in regulating spikelet morphogenesis in OE lines. We analyzed the expression of different class of MADS‐box genes in WT and *OsJAZ11* transgenics.

E‐class (*SEPALLATA*, *SEP*) genes in ABCDE model specify flower organ identity together with A‐, B‐, or C‐class proteins. Among the various E‐class MADS‐box genes (*OsMADS1/5/7/8/34*), we found significant downregulation of *OsMADS1* in OE lines as compared to WT (Figure S18). Notably, *osmads1* mutant displayed reiterative formation of glumes and spikelets within spikelets (Chen et al., 2006; Jeon et al., 2000). Expression of *OsMADS1* is also regulated by *OsMYC2* (Cai et al., 2014). This suggests that downregulation of *OsMADS1* in OE lines may be the direct consequence of reduced expression of *OsMYC2* in OE lines.

**FIGURE 6:**
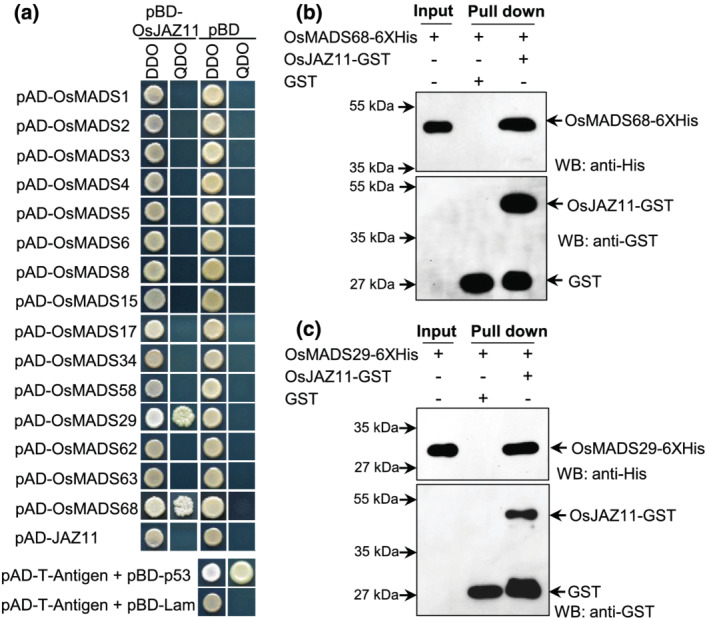
OsJAZ11 interacts with OsMADS29 and OsMADS68. (a) Yeast two‐hybrid interaction assays between pBD‐OsJAZ11 and pAD‐OsMADS. Yeast cells co‐transformed with AD (prey) and BD (bait) plasmids were spotted on DDO medium (SD‐Leu/‐Trp) and QDO medium (SD‐Leu/‐Trp/‐His/‐Ade). pBD and pAD represent empty BD (pGBKT7) and AD (pGADT7) vector, respectively. Interaction between pAD‐T‐Antigen and pBD‐p53 was used as positive control, whereas interaction between pAD‐T‐Antigen and pBD‐Lam was used as a negative control. (b) GST pull‐down assay showing interaction of OsJAZ11‐GST and OsMADS68‐6XHis. (c) GST pull‐down assay showing interaction of OsJAZ11‐GST and OsMADS29‐6XHis

B‐class MADS‐box genes (*OsMADS2*, *OsMADS4*, and *OsMADS16*), *OsMADS6*, and *OsMADS32* regulate lodicule development in rice. Expansion and shrinkage of lodicules are essential for opening and closing of hull. As OE lines exhibited open‐hull phenotypes, we also examined the expression of these genes in WT and transgenics. Notably, we found significant downregulation of *OsMADS2* in OE lines as compared to WT (Figure S18). In addition to this, we also found significant suppression of *OsMADS68* in OE lines compared to WT (Figure S18). Reduced expression of *OsMADS68* has been associated with reduced pollen viability (Liu et al., 2013). Notably, OsJAZ11 also showed interaction with OsMAD68 in yeast cells and in in vitro pull down assays (Figure 6a,b). We also investigated the interaction of other known MADS‐box genes (*OsMAD62/63*) involved in maintaining pollen viability; however, we did not observe any positive interactions between OsMADS62/63 and OsJAZ11.

Besides, examining the genes responsible for spikelet development, we also studied expression of *OsMADS29* implicated in seed development. Our expression analysis showed reduced expression of *OsMADS29* in Ri lines than WT (Figure S18). Interestingly, we also found protein–protein interaction between OsMADS29 and OsJAZ11 in yeast two‐hybrid assay (Figure 6a). Further, OsJAZ11‐GST could pull down OsMADS29‐6XHIS in in vitro pull‐down assay (Figure 6c). This further confirmed physical interaction of OsJAZ11 with OsMADS29. Apart from OsMADS29, we also investigated protein–protein interactions of several other MADS‐box genes with OsJAZ11; however, we did not find any positive interactions (Figure 6a).

### 3.8 *OsGW7* is suppressed in *OsJAZ11* overexpressing lines

**FIGURE 7:**
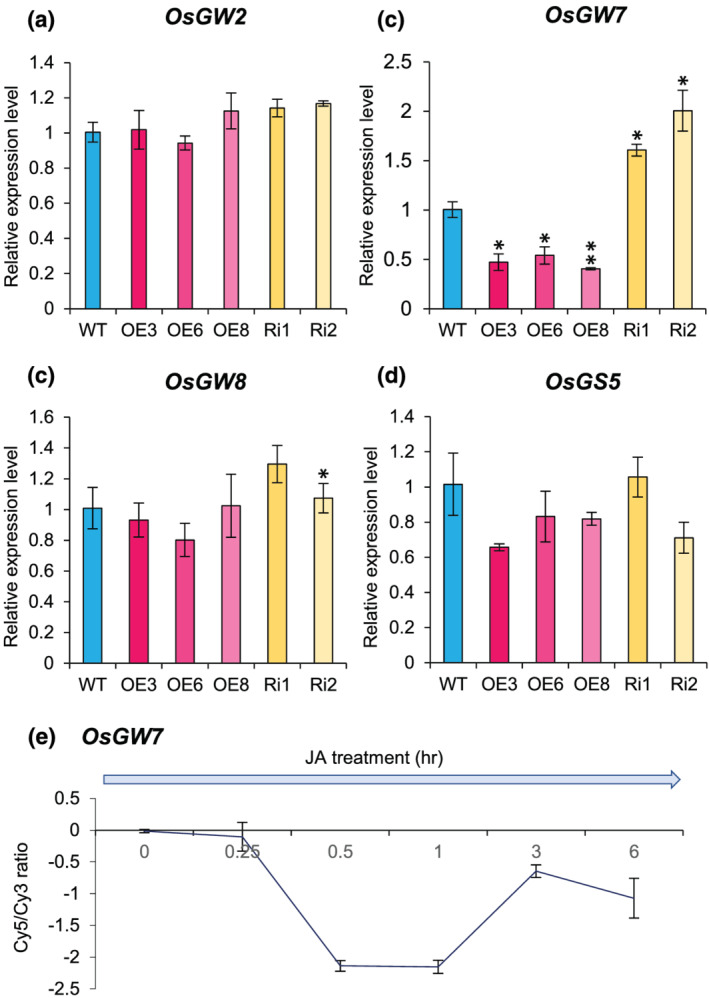
*OsJAZ11* overexpression suppressed expression of *OsGW7*. Relative expression levels of (a) *OsGW2*, (b) *OsGW7*, (c) *OsGW8*, and (d) *OsGS5* in P4 spikelets of WT, *OsJAZ11* OE, and Ri lines (*n* = 3). Error bar represents standard error. Significant differences between WT and transgenics were determined using Student's *t* test. *, **, and *** represent *p* values ≤.05, .01, and .001, respectively. (e) Expression profile of *OsGW7* in response to JA treatment. Expression pattern was retrieved from microarray database RiceXPro (Rice Expression Profile Database) version 3.0

In recent years, a number of genes or QTLs regulating grain shape and/or size have been identified. To investigate the expression of few of these key regulators (*OsGW2*, *OsGW7*, *OsGW8*, and *OsGS3*) in WT and *OsJAZ11* transgenics, we performed RT‐qPCR at heading stage (Figure 7). Strikingly, we found 1.8‐ to 2.5‐fold downregulation of *OsGW7* in OE lines compared to WT (Figure 7b). On the other hand, expression of *OsGW7* increased 1.6‐ to 2.0‐fold in Ri lines as compared to WT (Figure 7b). It is important to note that *OsGW7* encodes a homolog of *Arabidopsis* TRM protein (TONNEAU1‐recruiting motif) and is a known negative regulator of grain width in rice. Upregulation of *OsGW7* leads to formation of slender grains (Wang et al., 2015). Moreover, expression data retrieved from RiceXPro also revealed that *OsGW7* is a JA responsive gene. Exogenous application of JA results in reduced expression of *OsGW7* (Figure 7e). Thus, increased JA levels in *OsJAZ11* OE lines might also have suppressed expression of *OsGW7*. Together, these evidence point toward underlying functional roles of *OsGW7* in regulating grain width in OE lines. Therefore, we propose that *OsGW7* may be a key downstream regulator controlling grain size in *OsJAZ11* OE lines.

## 4 DISCUSSION

Rice is an important cereal crop that feeds about half of the world's population (Weng et al., 2008). As the global population is projected to touch the mark of 8.5 billion by 2030, there is immense pressure on breeding programs to increase rice yield. Seed length, width, and thickness are positively correlated with seed weight as well as yield (Tan et al., 2000). In the current study, we overexpressed and silenced a JAZ repressor, *OsJAZ11*, in rice. Our investigations uncovered that increased expression of *OsJAZ11* positively influence seed width and weight traits.

**FIGURE 8:**
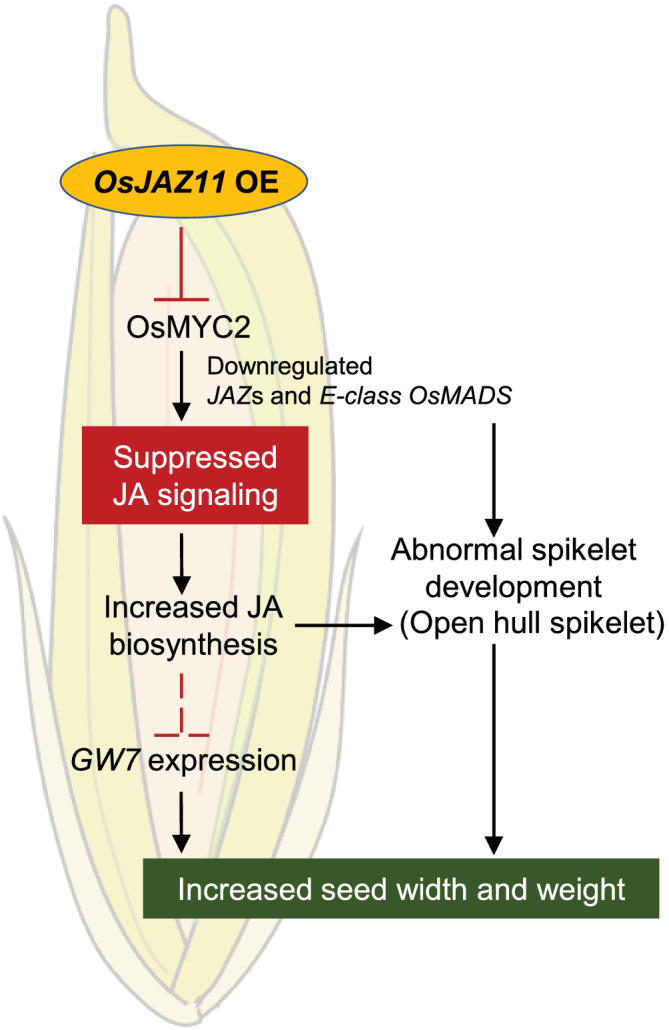
*OsJAZ11* regulates seed width and spikelet morphogenesis in rice. Schematic illustration of mode of action of *OsJAZ11*. Overexpression of *OsJAZ11* blocks *OsMYC2* regulated expression of other JAZ repressors and E‐class MADS. Suppressed JA signaling induces JA biosynthesis in spikelets (feedback loop) leading to abnormal spikelet morphogenesis. Increased hull size in overexpression lines allows wider seed growth. Overexpression of *OsJAZ11* also indirectly downregulates *OsGW7* leading to increased seed width

The potential size of the rice seed is physically restricted by the shape and size of the hull (Li & Li, 2016). Increase in hull size often leads to increase in seed weight (Fu et al., 2015; You et al., 2019). For instance, in *GW2* overexpressing lines, increased hull width increased grain width and weight (Song et al., 2007). Overexpression lines of *OsJAZ11* also showed wider hull phenotype as compared to WT (Figure S19). Therefore, *OsJAZ11* OE lines produced relatively wider seeds as compared to WT. Further analysis revealed suppressed expression of *GW7* in spikelets of OE lines. Notably, *GW7* is a key grain size regulator gene that negatively regulates rice grain width (Wang et al., 2015). Overexpression and silencing lines of *GW7* produced slender and wider grains, respectively. *GW7* promotes cell division in longitudinal direction in spikelet hulls which eventually leads to formation of slender grains. These evidence suggest that wider seed size of *OsJAZ11* OE lines could be the result of suppressed action of *GW7* in spikelets (Figure 8). As per earlier reports, another grain size regulator, *OsSPL16/GW8*, acts as a transcriptional repressor of *GW7* (Wang et al., 2015). *OsSPL16* encodes for a SBP (SQUAMOSA PROMOTER–BINDING PROTEIN)‐domain transcription factor and promotes cell proliferation in spikelet hulls leading to enhanced grain width (Wang et al., 2012). However, in our expression analysis, we did not observe any significant change in transcript level of *OsSPL16* in OE lines as compared to WT (Figure 7c). This indicates that *OsJAZ11* regulates *GW7* expression independent of *OsSPL16*.

Apart from enhanced seed width and weight, most of the spikelets in *OsJAZ11* OE lines also showed altered spikelet morphology. Previous study has shown spikelet defects in mutant of rice JAZ repressor, *OsJAZ1* (Cai et al., 2014). Similar to *osjaz1*, OE lines of *OsJAZ11* also exhibited extra glume‐like structures, extra spikelets within spikelets, and lemma‐like palea. Mutants of few other rice *JAZ* repressors also showed extra glume‐like structures and reduced number of floral organs (Hori et al., 2014). However, unlike these mutants, *OsJAZ11* OE lines displayed open hull phenotype and increased number of floral organs such as supernumerary stamens, multiple carpels, and lodicules. On the other hand, silencing of *OsJAZ11* did not produce any spikelet defects. Analogous to OsJAZ1, OsJAZ11 was found to form protein complexes with common components of JA machinery including OsMYC2 transcription factor (master regulator of JA‐response) and OsNINJA1 (Novel INteractor of JAZ) (Pandey et al., 2021). OsJAZ11 also heterodimerizes with other rice JAZ repressors including OsJAZ1. Thus, despite sharing common JA signaling pathways, different JAZ repressors seem to exhibit functional diversity in regulating rice reproductive development. Previously, some other JAZ repressors such as OsJAZ8 have also been shown to heterodimerize with other JAZs to regulate JA signaling pathways in rice (Yamada et al., 2012).

In the present study, we report interaction of some important MADS transcription factor (OsMADS68 and OsMAD29) with OsJAZ11. OsMADS68 and OsMADS29 have been reported for their essential roles in maintaining pollen viability and seed development, respectively (Liu et al., 2013; Nayar et al., 2013; Yin & Xue, 2012). Additionally, we found reduced expression of *OsMADS1* in OE lines. This could be a result of decreased expression of its regulator, *OsMYC2* in OE lines. Reduced expression of *OsMADS1* and *OsMYC2* was also reported in *osjaz1* mutant with extra glume phenotype (Cai et al., 2014). *LHS1/OsMADS1* is an E class gene and is essential for specification of lemma and palea. Mutations in *OsMADS1* lead to formation of leaf‐like lemma and palea (Prasad et al., 2005). *OsMADS1* is responsible for spikelet‐to‐floret meristem transition and meristem maintenance (Khanday et al., 2013). Moreover, mutant of *OsMADS1* also exhibited open‐hull phenotype at early stages of panicle development, an event very similar to *OsJAZ11* OE lines (Jeon et al., 2000). Thus, reduced expression of *OsMADS1* in OE lines may have directly or indirectly led to open‐hull phenotype in OE lines. Recent studies have also reported crucial roles of *OsMADS1* in regulating grain size (Huang et al., 2020; Liu et al., 2018; Yu et al., 2018). Hence, altered expression of *OsMADS1* in *OsJAZ11* OE lines might have influenced spikelet and seed development in OE lines.

As OsJAZ11 is a JA signaling repressor, overexpression of *OsJAZ11* suppressed JA signaling in OE lines. This is evident from downregulation of JA signaling genes such as *OsMYC2* and other *JAZ* repressors. We further found significantly high JA levels in OE lines. This suggests that suppressed JA signaling has induced JA biosynthesis in reproductive organs of OE lines in a feedback loop (Figure 8). Higher JA levels has also been associated with increased number of floral organs and reduced rice yield. For example, higher JA levels in overexpression lines of *OsJMT1* (JA carboxyl methyltransferase) led to formation of 5–10 stamens, multiple pistils, and compound ovary with multiple stigma branches (Kim et al., 2009). JA levels in spikelet tissues also regulate optimal opening and closing of flower (Xiao et al., 2014). Exogenous application of MeJA induces spikelet opening in rice, and the number of opened florets is directly correlated with the concentration of applied MeJA (Zeng et al., 1999). These lines of evidence suggest that abnormal spikelet phenotypes in *OsJAZ11* OE lines may be the effect of enhanced JA levels in OE lines. In addition, we found significantly higher expression of *OsJAZ11* in early panicle developmental stages. Previous studies have also reported strong expression of key seed size regulators in early panicle development stages (You et al., 2019). Taken together, our findings suggest critical roles of *OsJAZ11* in determining seed width and weight. However, in future, it would be interesting to dissect the complete signaling cascade that determines final seed size in *OsJAZ11* OE lines. The series of molecular events that regulate *GW7* expression in OE lines also remains to be elucidated. Nevertheless, our investigations provide new insights about JA signaling mechanisms that modulate seed development and yield in rice. Besides, the present study also adds to our knowledge for improving high‐yielding elite rice genotypes through genetic engineering.

## CONFLICT OF INTEREST

The authors declare no conflict of interest.

## AUTHOR CONTRIBUTIONS

PM, AKT, and JG conceived the study. PM, BKP, LV, AP, APS, SS, and NM conducted the experiments. PM, BKP, SKP, JG, MJB, and AKT analyzed the data, interpreted results, and discussed outcomes. PM, BKP, and SKP wrote the article. SKP, JG, MJB, and AKT edited the manuscript. All authors read and approved the final version of the manuscript.

## Supporting information


**Figure S1.** Expression of *OsJAZ11* in panicle and seed development stages*.*
**Figure S2.** Expression patterns of *OsJAZ11*.
**Figure S3.** Raising of *OsJAZ11* translational reporters.
**Figure S4.** Relative expression levels of *OsJAZ11* in WT, OE and Ri transgenics.
**Figure S5.** Effect of *OsJAZ11* on seed length.
**Figure S6.** Seed phenotypes of *OsJAZ11* transgenics in T2 generation.
**Figure S7.** Effect of *OsJAZ11* on panicle length.
**Figure S8.** Panicle phenotype of *OsJAZ11* transgenics.
**Figure S9.** Effect of *OsJAZ11* on seed number.
**Figure S10.** Effect of *OsJAZ11* on percentage of filled seeds per panicle.
**Figure S11.** Effect of *OsJAZ11* on yield.
**Figure S12.** Spikelet phenotype of *OsJAZ11* transgenics.
**Figure S13.** Spikelet phenotype of *OsJAZ11* transgenics before heading.
**Figure S14.** Pollen viability of *OsJAZ11* overexpression lines.
**Figure S15.** Pollen viability of WT and *OsJAZ11* transgenics.
**Figure S16.** OsJAZ11 heterodimerizes with other JAZ protein.
**Figure S17.** OsJAZ11 interacts with OsJAZ1.
**Figure S18.** Effect of *OsJAZ11* on expression of MADS transcription factors.
**Figure S19.** Overexpression of *OsJAZ11* leads to widening of hulls.

**Table S1.** List of primers used in the study

**Table S2.** Proportion of open hull spikelets among total defective spikelets in *OsJAZ11* OE lines

**Table S3.** Number of stamens, carpels and extra glume‐like structures observed in *OsJAZ11* OE lines

## Data Availability

Details of all data associated with the manuscript are available in the main text or in the supporting information.

## References

[pld3401-bib-0001] Ali, M. S. , & Baek, K. H. (2020). Jasmonic acid signaling pathway in response to abiotic stresses in plants. International Journal of Molecular Sciences, 21, 621. 10.3390/ijms21020621 PMC7013817 31963549

[pld3401-bib-0002] Cai, Q. , Yuan, Z. , Chen, M. , Yin, C. , Luo, Z. , Zhao, X. , Liang, W. , Hu, J. , & Zhang, D. (2014). Jasmonic acid regulates spikelet development in rice. Nature Communications, 5(1), 3476. 10.1038/ncomms4476 24647160

[pld3401-bib-0003] Callens, C. , Tucker, M. R. , Zhang, D. , & Wilson, Z. A. (2018). Dissecting the role of MADS‐box genes in monocot floral development and diversity. Journal of Experimental Botany, 69(10), 2435–2459. 10.1093/jxb/ery086 29718461

[pld3401-bib-0004] Che, R. H. , Tong, H. N. , Shi, B. H. , Liu, Y. Q. , Fang, S. R. , Liu, D. P. , Xiao, Y. H. , Hu, B. , Liu, L. C. , Wang, H. R. , Zhao, M. F. , & Chu, C. C. (2016). Control of grain size and rice yield by GL2‐mediated brassinosteroid response. Nature Plants, 2, 15195–15201. 10.1038/nplants.2015.195 27250747

[pld3401-bib-0005] Chen, Z. X. , Wu, J. G. , Ding, W. N. , Chen, H. M. , Wu, P. , & Shi, C. H. (2006). Morphogenesis and molecular basis on *naked seed rice*, a novel homeotic mutation of *OsMADS1* regulating transcript level of AP3 homologue in rice. Planta, 223(5), 882–890. 10.1007/s00425-005-0141-8 16254725

[pld3401-bib-0006] Chini, A. , Fonseca, S. , Fernández, G. , Adie, B. , Chico, J. M. , Lorenzo, O. , García‐Casado, G. , López‐Vidriero, I. , Lozano, F. M. , Ponce, M. R. , Micol, J. L. , & Solano, R. (2007). The JAZ family of repressors is the missing link in jasmonate signalling. Nature, 448(7154), 666–671. 10.1038/nature06006 17637675

[pld3401-bib-0007] Chongloi, G. L. , Prakash, S. , & Vijayraghavan, U. (2019). Rice shoot and floral meristem development: An overview of developmental regulators of meristem maintenance and organ identity. Journal of Experimental Botany, 70(6), 1719–1736. 10.1093/jxb/erz046 30753578

[pld3401-bib-0008] Fan, C. , Xing, Y. , Mao, H. , Lu, T. , Han, B. , Xu, C. , Li, X. , & Zhang, Q. (2006). *GS3*, a major QTL for grain length and weight and minor QTL for grain width and thickness in rice, encodes a putative transmembrane protein. Theoretical and Applied Genetics, 112(6), 1164–1171. 10.1007/s00122-006-0218-1 16453132

[pld3401-bib-0009] Fu, J. R. , Zhu, L. X. , Sun, X. T. , Zhou, D. H. , Ouyang, L. J. , Bian, J. M. , He, H. H. , & Xu, J. (2015). Genetic analysis of grain shape and weight after cutting rice husk. Genetics and Molecular Research, 14(4), 17739–17748. 10.4238/2015.December.21.47 26782419

[pld3401-bib-0010] Hori, Y. , Kurotani, K. , Toda, Y. , Hattori, T. , & Takeda, S. (2014). Overexpression of the JAZ factors with mutated jas domains causes pleiotropic defects in rice spikelet development. Plant Signaling and Behavior, 9(10), e970414. 10.4161/15592316.2014.970414 25482801 PMC4623050

[pld3401-bib-0011] Hu, J. , Wang, Y. , Fang, Y. , Zeng, L. , Xu, J. , Yu, H. , Shi, Z. , Pan, J. , Zhang, D. , Kang, S. , Zhu, L. , Dong, G. , Guo, L. , Zeng, D. , Zhang, G. , Xie, L. , Xiong, G. , Li, J. , & Qian, Q. (2015). A rare allele of GS2 enhances grain size and grain yield in rice. Molecular Plant, 8(10), 1455–1465. 10.1016/j.molp.2015.07.002 26187814

[pld3401-bib-0012] Huang, H. , Liu, B. , Liu, L. , & Song, S. (2017). Jasmonate action in plant growth and development. Journal of Experimental Botany, 68, 1349–1359. 10.1093/jxb/erw495 28158849

[pld3401-bib-0013] Huang, Y. , Bai, X. , Cheng, N. , Xiao, J. , Li, X. , & Xing, Y. (2020). *Wide grain 7* increases grain width by enhancing H3K4me3 enrichment in the *OsMADS1* promoter in rice (*Oryza sativa* L.). The Plant Journal, 102(3), 517–528. 10.1111/tpj.14646 31830332

[pld3401-bib-0014] Ishimaru, K. , Hirotsu, N. , Madoka, Y. , Murakami, N. , Hara, N. , Onodera, H. , Kashiwagi, T. , Ujiie, K. , Shimizu, B. , Onishi, A. , Miyagawa, H. , & Katoh, E. (2013). Loss of function of the IAA‐glucose hydrolase gene *TGW6* enhances rice grain weight and increases yield. Nature Genetics, 45(6), 707–711. 10.1038/ng.2612 23583977

[pld3401-bib-0015] Jang, G. , Yoon, Y. , & Choi, Y. D. (2019). Jasmonic acid modulates xylem development by controlling expression of *PIN‐FORMED 7* . Plant Signaling and Behavior, 14(9), 1637664. 10.1080/15592324.2019.1637664 31264505 PMC6768215

[pld3401-bib-0016] Jeon, J. S. , Jang, S. , Lee, S. , Nam, J. , Kim, C. , Lee, S. H. , Chung, Y. Y. , Kim, S. R. , Lee, Y. H. , Cho, Y. G. , & An, G. (2000). *Leafy hull sterile1* is a homeotic mutation in a rice MADS box gene affecting rice flower development. The Plant Cell, 12(6), 871–884. 10.1105/tpc.12.6.871 10852934 PMC149090

[pld3401-bib-0017] Khanday, I. , Yadav, S. R. , & Vijayraghavan, U. (2013). Rice *LHS1/OsMADS1* controls floret meristem specification by coordinated regulation of transcription factors and hormone signaling pathways. Plant Physiology, 161(4), 1970–1983. 10.1104/pp.112.212423 23449645 PMC3613468

[pld3401-bib-0018] Kim, E. H. , Kim, Y. S. , Park, S. H. , Koo, Y. J. , Choi, Y. D. , Chung, Y. Y. , Lee, I. J. , & Kim, J. K. (2009). Methyl jasmonate reduces grain yield by mediating stress signals to alter spikelet development in rice. Plant Physiology, 149(4), 1751–1760. 10.1104/pp.108.134684 19211695 PMC2663756

[pld3401-bib-0019] Li, N. , & Li, Y. (2016). Signaling pathways of seed size control in plants. Current Opinion in Plant Biology, 33, 23–32. 10.1016/j.pbi.2016.05.008 27294659

[pld3401-bib-0020] Li, Y. , Fan, C. , Xing, Y. , Jiang, Y. , Luo, L. , Sun, L. , Shao, D. , Xu, C. , Li, X. , Xiao, J. , He, Y. , & Zhang, Q. (2011). Natural variation in *GS5* plays an important role in regulating grain size and yield in rice. Nature Genetics, 43, 1266–1269. 10.1038/ng.977 22019783

[pld3401-bib-0021] Lin, H. F. , Xiong, J. , Zhou, H. M. , Chen, C. M. , Lin, F. Z. , Xu, X. M. , Oelmüller, R. , Xu, W. F. , & Yeh, K. W. (2019). Growth promotion and disease resistance induced in *Anthurium* colonized by the beneficial root endophyte *Piriformospora indica* . BMC Plant Biology, 19, 40. 10.1186/s12870-019-1649-6 30678653 PMC6346537

[pld3401-bib-0022] Liu, Q. , Han, R. , Wu, K. , Zhang, J. , Ye, Y. , Wang, S. , Chen, J. , Pan, Y. , Li, Q. , Xu, X. , Zhou, J. , Tao, D. , Wu, Y. , & Fu, X. (2018). G‐protein βγ subunits determine grain size through interaction with MADS‐domain transcription factors in rice. Nature Communications, 9(1), 852. 10.1038/s41467-018-03047-9 PMC5829230 29487282

[pld3401-bib-0023] Liu, Y. , Cui, S. , Wu, F. , Yan, S. , Lin, X. , Du, X. , Chong, K. , Schilling, S. , Theißen, G. , & Meng, Z. (2013). Functional conservation of MIKC*‐type MADS box genes in *Arabidopsis* and rice pollen maturation. The Plant Cell, 25(4), 1288–1303. 10.1105/tpc.113.110049 23613199 PMC3663268

[pld3401-bib-0024] Mehra, P. , & Giri, J. (2016). Rice and chickpea GDPDs are preferentially influenced by low phosphate and CaGDPD1 encodes an active glycerophosphodiester phosphodiesterase enzyme. Plant Cell Reports, 35(8), 1699–1717. 10.1007/s00299-016-1984-0 27108120

[pld3401-bib-0025] Mehra, P. , Pandey, B. K. , & Giri, J. (2017). Improvement in phosphate acquisition and utilization by a secretory purple acid phosphatase (OsPAP21b) in rice. Plant Biotechnology Journal, 15(8), 1054–1067. 10.1111/pbi.12699 28116829 PMC5506657

[pld3401-bib-0026] Mehra, P. , Pandey, B. K. , Verma, L. , & Giri, J. (2019). A novel glycerophosphodiester phosphodiesterase improves phosphate deficiency tolerance in rice. Plant, Cell and Environment, 42, 1167–1179. 10.1111/pce.13459 30307043

[pld3401-bib-0027] Nayar, S. , Sharma, R. , Tyagi, A. K. , & Kapoor, S. (2013). Functional delineation of rice MADS29 reveals its role in embryo and endosperm development by affecting hormone homeostasis. Journal of Experimental Botany, 64(14), 4239–4253. 10.1093/jxb/ert231 23929654 PMC3808311

[pld3401-bib-0028] Pandey, B. K. , Mehra, P. , Verma, L. , Bhadouria, J. , & Giri, J. (2017). OsHAD1, a haloacid dehalogenase‐like APase, enhances phosphate accumulation. Plant Physiology, 174(4), 2316–2332. 10.1104/pp.17.00571 28637831 PMC5543963

[pld3401-bib-0029] Pandey, B. K. , Verma, L. , Prusty, A. , Singh, A. P. , Bennett, M. J. , Tyagi, A. K. , Giri, J. , & Mehra, P. (2021). *OsJAZ11* regulates phosphate starvation responses in rice. Planta, 254, 1–6. 10.1007/s00425-021-03657-6 34143292 PMC8213676

[pld3401-bib-0030] Pauwels, L. , Barbero, G. F. , Geerinck, J. , Tilleman, S. , Grunewald, W. , Pérez, A. C. , Chico, J. M. , Bossche, R. V. , Sewell, J. , Gil, E. , García‐Casado, G. , Witters, E. , Inzé, D. , Long, J. A. , De Jaeger, G. , Solano, R. , & Goossens, A. (2010). NINJA connects the co‐repressor TOPLESS to jasmonate signalling. Nature, 464(7289), 788–791. 10.1038/nature08854 20360743 PMC2849182

[pld3401-bib-0031] Pauwels, L. , & Goossens, A. (2011). The JAZ proteins: A crucial interface in the jasmonate signaling cascade. The Plant Cell, 23(9), 3089–3100. 10.1105/tpc.111.089300 21963667 PMC3203442

[pld3401-bib-0032] Prasad, K. , Parameswaran, S. , & Vijayraghavan, U. (2005). *OsMADS1*, a rice MADS‐box factor, controls differentiation of specific cell types in the lemma and palea and is an early‐acting regulator of inner floral organs. The Plant Journal, 43(6), 915–928. 10.1111/j.1365-313X.2005.02504.x 16146529

[pld3401-bib-0033] Qi, P. , Lin, Y. S. , Song, X. J. , Shen, J. B. , Huang, W. , Shan, J. X. , Zhu, M. Z. , Jiang, L. , Gao, J. P. , & Lin, H. X. (2012). The novel quantitative trait locus *GL3.1* controls rice grain size and yield by regulating cyclin‐T1;3. Cell Research, 22(12), 1666–1680. 10.1038/cr.2012.151 23147796 PMC3515756

[pld3401-bib-0034] Ranjan, R. , Khurana, R. , Malik, N. , Badoni, S. , Parida, S. K. , Kapoor, S. , & Tyagi, A. K. (2017). *bHLH142* regulates various metabolic pathway‐related genes to affect pollen development and anther dehiscence in rice. Scientific Reports, 7(1), 43397. 10.1038/srep43397 28262713 PMC5338287

[pld3401-bib-0035] Sato, Y. , Antonio, B. A. , Namiki, N. , Takehisa, H. , Minami, H. , Kamatsuki, K. , Sugimoto, K. , Shimizu, Y. , Hirochika, H. , & Nagamura, Y. (2010). RiceXPro: A platform for monitoring gene expression in *japonica* rice grown under natural field conditions. Nucleic Acids Research, 39, D1141–D1148. 21045061 10.1093/nar/gkq1085 PMC3013682

[pld3401-bib-0036] Seck, P. A. , Diagne, A. , Mohanty, S. , & Wopereis, M. C. S. (2012). Crops that feed the world 7: Rice. Food Security, 4(1), 7–24. 10.1007/s12571-012-0168-1

[pld3401-bib-0037] Seo, J. S. , Joo, J. , Kim, M. J. , Kim, Y. K. , Nahm, B. H. , Song, S. I. , Cheong, J. J. , Lee, J. S. , Kim, J. K. , & Choi, Y. D. (2011). OsbHLH148, a basic helix‐loop‐helix protein, interacts with OsJAZ proteins in a jasmonate signaling pathway leading to drought tolerance in rice. The Plant Journal, 65, 907–921. 10.1111/j.1365-313X.2010.04477.x 21332845

[pld3401-bib-0038] Singh, A. P. , Pandey, B. K. , Deveshwar, P. , Narnoliya, L. , Parida, S. K. , & Giri, J. (2015). JAZ repressors: Possible involvement in nutrients deficiency response in rice and chickpea. Frontiers in Plant Science, 6, 975. 10.3389/fpls.2015.00975 26617618 PMC4639613

[pld3401-bib-0039] Singh, A. P. , Pandey, B. K. , Mehra, P. , Heitz, T. , & Giri, J. (2020). *OsJAZ9* overexpression modulates jasmonic acid biosynthesis and potassium deficiency responses in rice. Plant Molecular Biology, 104(4–5), 397–410. 10.1007/s11103-020-01047-2 32803476

[pld3401-bib-0040] Song, X. J. , Huang, W. , Shi, M. , Zhu, M. Z. , & Lin, H. X. (2007). A QTL for rice grain width and weight encodes a previously unknown RING‐type E3 ubiquitin ligase. Nature Genetics, 39(5), 623–630. 10.1038/ng2014 17417637

[pld3401-bib-0041] Song, X. J. , Kuroha, T. , Ayano, M. , Furuta, T. , Nagai, K. , Komeda, N. , Segami, S. , Miura, K. , Ogawa, D. , Kamura, T. , Suzuki, T. , Higashiyama, T. , Yamasaki, M. , Mori, H. , Inukai, Y. , Wu, J. Z. , Kitano, H. , Sakakibara, H. , Jacobsen, E. S. , & Ashikari, M. (2015). Rare allele of a previously unidentified histone H4 acetyltransferase enhances grain weight, yield, and plant biomass in rice. Proceedings of the National Academy of Sciences of the United States of America, 112, 76–81. 10.1073/pnas.1421127112 25535376 PMC4291654

[pld3401-bib-0042] Staswick, P. E. (2008). JAZing up jasmonate signaling. Trends in Plant Science, 13, 66–71. 10.1016/j.tplants.2007.11.011 18261950

[pld3401-bib-0043] Tan, Y. F. , Xing, Y. Z. , Li, J. X. , Yu, S. B. , Xu, C. G. , & Zhang, Q. (2000). Genetic bases of appearance quality of rice grains in Shanyou 63, an elite rice hybrid. Theoretical and Applied Genetics, 101(5–6), 823–829. 10.1007/s001220051549 22665200

[pld3401-bib-0044] Trang Nguyen, H. , Thi Mai To, H. , Lebrun, M. , Bellafiore, S. , & Champion, A. (2019). Jasmonates‐the master regulator of rice development, adaptation and defense. Plants, 8(9), 339. 10.3390/plants8090339 PMC6784130 31505882

[pld3401-bib-0045] Wang, J. , Song, L. , Gong, X. , Xu, J. , & Li, M. (2020). Functions of jasmonic acid in plant regulation and response to abiotic stress. International Journal of Molecular Sciences, 21(4), 1446. 10.3390/ijms21041446 PMC7073113 32093336

[pld3401-bib-0046] Wang, S. , Li, S. , Liu, Q. , Wu, K. , Zhang, J. , Wang, S. , Wang, Y. , Chen, X. , Zhang, Y. , Gao, C. , Wang, F. , Huang, H. , & Fu, X. (2015). The *OsSPL16‐GW7* regulatory module determines grain shape and simultaneously improves rice yield and grain quality. Nature Genetics, 47(8), 949–954. 10.1038/ng.3352 26147620

[pld3401-bib-0047] Wang, S. , Wu, K. , Yuan, Q. , Liu, X. , Liu, Z. , Lin, X. , Zeng, R. Z. , Zhu, H. , Dong, G. , Qian, Q. , Zhang, G. , & Fu, X. (2012). Control of grain size, shape and quality by *OsSPL16* in rice. Nature Genetics, 44(8), 950–954. 10.1038/ng.2327 22729225

[pld3401-bib-0049] Wasternack, C. , & Hause, B. (2013). Jasmonates: Biosynthesis, perception, signal transduction and action in plant stress response, growth and development. An update to the 2007 review in annals of botany. Annals of Botany, 111(6), 1021–1058. 10.1093/aob/mct067 23558912 PMC3662512

[pld3401-bib-0050] Weng, J. , Gu, S. , Wan, X. , Gao, H. , Guo, T. , Su, N. , Lei, C. , Zhang, X. , Cheng, Z. , Guo, X. , Wang, J. , Jiang, L. , Zhai, H. , & Wan, J. (2008). Isolation and initial characterization of *GW5*, a major QTL associated with rice grain width and weight. Cell Research, 18, 1199–1209. 10.1038/cr.2008.307 19015668

[pld3401-bib-0051] Wu, H. , Ye, H. , Yao, R. , Zhang, T. , & Xiong, L. (2015). OsJAZ9 acts as a transcriptional regulator in jasmonate signaling and modulates salt stress tolerance in rice. Plant Science, 232, 1–12. 10.1016/j.plantsci.2014.12.010 25617318

[pld3401-bib-0052] Xiao, Y. , Chen, Y. , Charnikhova, T. , Mulder, P. P. , Heijmans, J. , Hoogenboom, A. , Agalou, A. , Michel, C. , Morel, J. B. , Dreni, L. , Kater, M. M. , Bouwmeester, H. , Wang, M. , Zhu, Z. , & Ouwerkerk, P. B. (2014). *OsJAR1* is required for JA‐regulated floret opening and anther dehiscence in rice. Plant Molecular Biology, 86(1–2), 19–33. 10.1007/s11103-014-0212-y 24947835

[pld3401-bib-0053] Yamada, S. , Kano, A. , Tamaoki, D. , Miyamoto, A. , Shishido, H. , Miyoshi, S. , Taniguchi, S. , Akimitsu, K. , & Gomi, K. (2012). Involvement of OsJAZ8 in jasmonate‐induced resistance to bacterial blight in rice. Plant Cell Physiology, 53(12), 2060–2072. 10.1093/pcp/pcs145 23104764

[pld3401-bib-0054] Yan, J. , Zhang, C. , Gu, M. , Bai, Z. , Zhang, W. , Qi, T. , Cheng, Z. , Peng, W. , Luo, H. , Nan, F. , Wang, Z. , & Xie, D. (2009). The *Arabidopsis* CORONATINE INSENSITIVE1 protein is a jasmonate receptor. The Plant Cell, 21(8), 2220–2236. 10.1105/tpc.109.065730 19717617 PMC2751961

[pld3401-bib-0055] Ye, H. , Du, H. , Tang, N. , Li, X. , & Xiong, L. (2009). Identification and expression profiling analysis of TIFY family genes involved in stress and phytohormone responses in rice. Plant Molecular Biology, 71, 291–305. 10.1007/s11103-009-9524-8 19618278

[pld3401-bib-0056] Yin, L.‐L. , & Xue, H.‐W. (2012). The MADS29 transcription factor regulates the degradation of the nucellus and the nucellar projection during rice seed development. The Plant Cell, 24(3), 1049–1065. 10.1105/tpc.111.094854 22408076 PMC3336122

[pld3401-bib-0057] Ying, J. Z. , Ma, M. , Bai, C. , Huang, X. H. , Liu, J. L. , & Song, X. J. (2018). *TGW3*, a major QTL that negatively modulates grain length and weight in rice. Molecular Plant, 11(5), 750–753. 10.1016/j.molp.2018.03.007 29567450

[pld3401-bib-0058] You, X. , Zhu, S. , Zhang, W. , Zhang, J. , Wang, C. , Jing, R. , Chen, W. , Wu, H. , Cai, Y. , Feng, Z. , Hu, J. , Yan, H. , Kong, F. , Zhang, H. , Zheng, M. , Ren, Y. , Lin, Q. , Cheng, Z. , Zhang, X. , … Wan, J. (2019). OsPEX5 regulates rice spikelet development through modulating jasmonic acid biosynthesis. New Phytologist, 224, 712–724. 10.1111/nph.16037 31264225

[pld3401-bib-0059] Yu, J. , Miao, J. , Zhang, Z. , Xiong, H. , Zhu, X. , Sun, X. , Pan, Y. , Liang, Y. , Zhang, Q. , Abdul Rehman, R. M. , Li, J. , Zhang, H. , & Li, Z. (2018). Alternative splicing of *OsLG3b* controls grain length and yield in japonica rice. Plant Biotechnology Journal, 16(9), 1667–1678. 10.1111/pbi.12903 PMC6097128 29479793

[pld3401-bib-0060] Yuan, Z. , & Zhang, D. (2015). Roles of Jasmonate signalling in plant inflorescence and flower development. Current Opinion in Plant Biology, 27, 44–51. 10.1016/j.pbi.2015.05.024 26125498

[pld3401-bib-0061] Zeng, X. , Zhou, X. , Zhang, W. , Murofushi, N. , Kitahara, T. , & Kamuro, Y. (1999). Opening of rice floret in rapid response to methyl jasmonate. Journal of Plant Growth Regulation, 18(4), 153–158. 10.1007/PL00007063 10688703

[pld3401-bib-0062] Zhang, W. , Sun, P. , He, Q. , Shu, F. , Wang, J. , & Deng, H. (2013). Fine mapping of *GS2*, a dominant gene for big grain rice. The Crop Journal, 2, 160–165. 10.1016/j.cj.2013.10.003

