# Supplementary material for: OsJAZ11 regulates spikelet and seed development in rice
Source: Plant Direct. 2022 May 10;6(5):e401. doi: 10.1002/pld3.401 (PMC9090556; doi:10.1002/pld3.401)
Supplement: Supplementary file 1 — Figure S1. Expression of OsJAZ11 in panicle and seed development stages. Figure S2. Expression patterns of OsJAZ11. Figure S3. Raising of OsJAZ11 translational reporters. Figure S4. Relative expression levels of OsJAZ11 in WT, OE and Ri transgenics. Figure S5. Effect of OsJAZ11 on seed length. Figure S6. Seed phenotypes of OsJAZ11 transgenics in T2 generation. Figure S7. Effect of OsJAZ11 on panicle length. Figure S8. Panicle phenotype of OsJAZ11 transgenics. Figure S9. Effect of OsJAZ11 on seed number. Figure S10. Effect of OsJAZ11 on percentage of filled seeds per panicle. Figure S11. Effect of OsJAZ11 on yield. Figure S12. Spikelet phenotype of OsJAZ11 transgenics. Figure S13. Spikelet phenotype of OsJAZ11 transgenics before heading. Figure S14. Pollen viability of OsJAZ11 overexpression lines. Figure S15. Pollen viability of WT and OsJAZ11 transgenics. Figure S16. OsJAZ11 heterodimerizes with other JAZ protein. Figure S17. OsJAZ11 interacts with OsJAZ1. Figure S18. Effect of OsJAZ11 on expression of MADS transcription factors. Figure S19. Overexpression of OsJAZ11 leads to widening of hulls. [file PLD3-6-e401-s001.pdf]

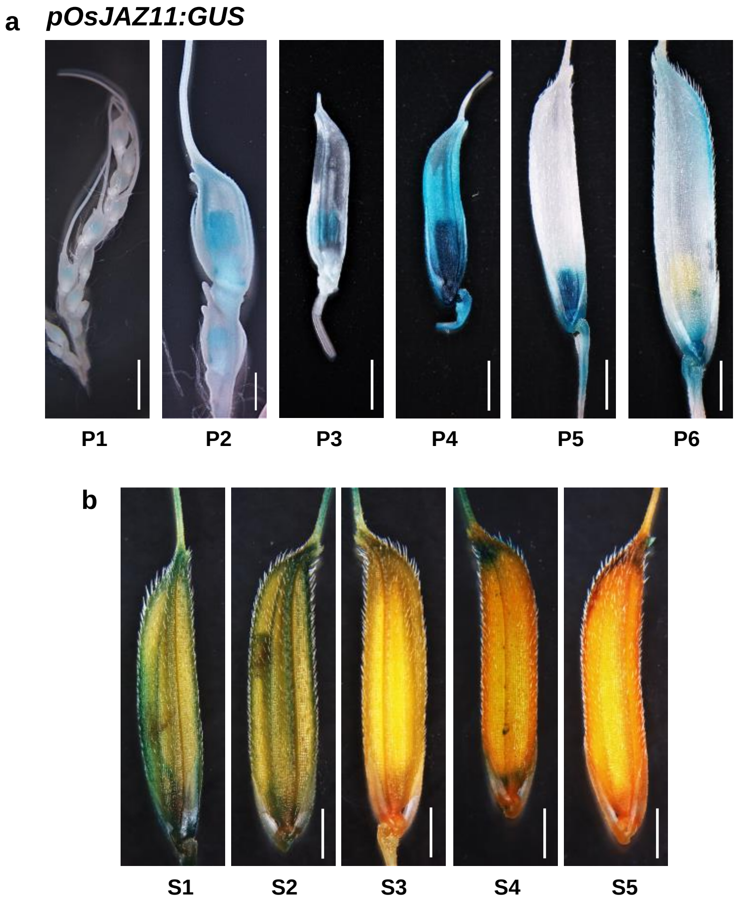

Figure S1. Expression of *OsJAZ11* in panicle and seed development stages. GUS staining of spikelets expressing *pOsJAZ11:GUS*. Histochemical staining was performed in different (a) panicle developmental stages (P1-P6) and (b) seed developmental stages (S1-S5). Bar = 2 mm.

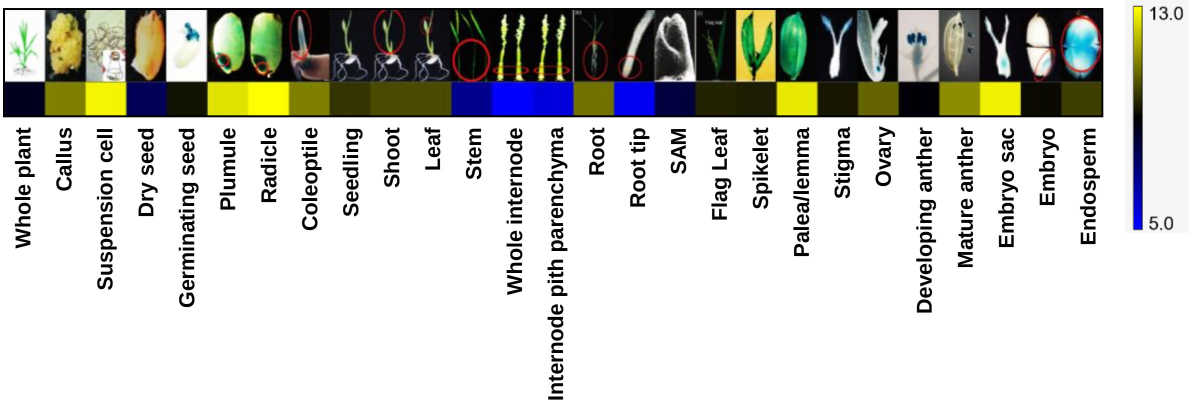

Figure S2. Expression patterns of *OsJAZ11*. Expression patterns of *OsJAZ11* in different tissues retrieved from rice microarray database, RiceXPro (The Rice Expression Profile Database).

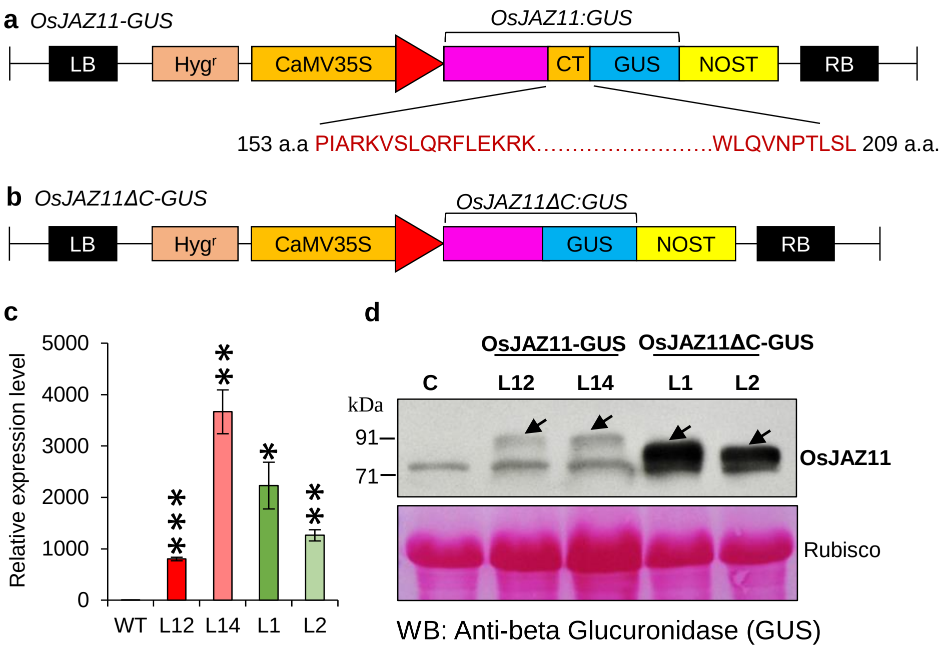

Figure S3. Raising of *OsJAZ11* translational reporters. Schematic illustration of (a) *OsJAZ11-GUS* and (b) *OsJAZ11ΔC-GUS* translational fusion constructs in pCAMBIA1301. In *OsJAZ11ΔC-GUS*, 57 a.a. (153- 209 a.a.) containing Jas domain was deleted from OsJAZ11 ORF (Pandey et al., 2021). (c) Relative expression levels of *OsJAZ11* in *OsJAZ11-GUS* (L12, L14) and *OsJAZ11ΔC-GUS* (L1, L2) transgenics with respect to WT. Expression levels were measured in spikelets of P4 stages (n=3). Error bar represents standard error. Significant differences between WT and transgenics were determined using Student's *t*-test. Asterisks; *, ** and *** represent *p* values, ≤ 0.05, 0.01 and 0.001, respectively. (d) Immunoblot of WT, OsJAZ11-GUS and OsJAZ11ΔC-GUS proteins with anti-β Glucuronidase antibody. 40 µg proteins from P4 spikelet tissues was loaded in each well. Black arrow indicates OsJAZ11-GUS/OsJAZ11ΔC-GUS proteins. Ponceau S staining of Rubisco was used to monitor equal loading of protein samples.

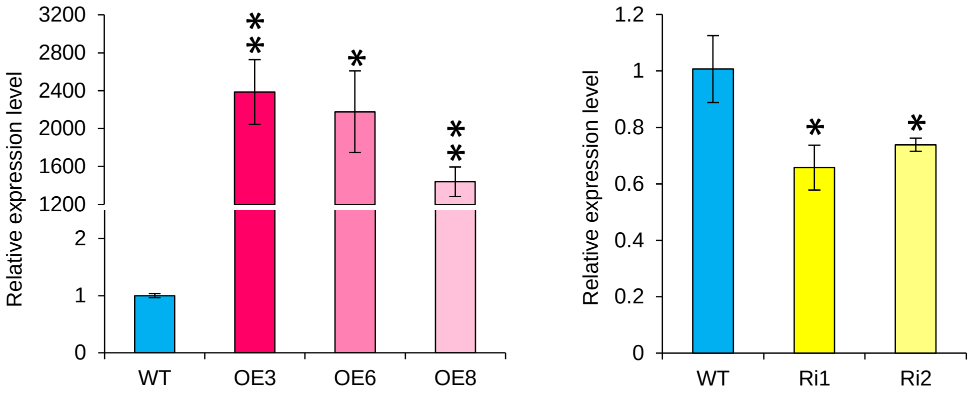

Figure S4. Relative expression levels of *OsJAZ11* in WT, OE and Ri transgenics. Expression levels were measured in spikelets of P4 stages (n=3). Error bar represents standard error. Significant differences between WT and transgenics were determined using Student's *t*-test. Asterisks; * and ** indicate *p* values, ≤ 0.05 and 0.01, respectively.

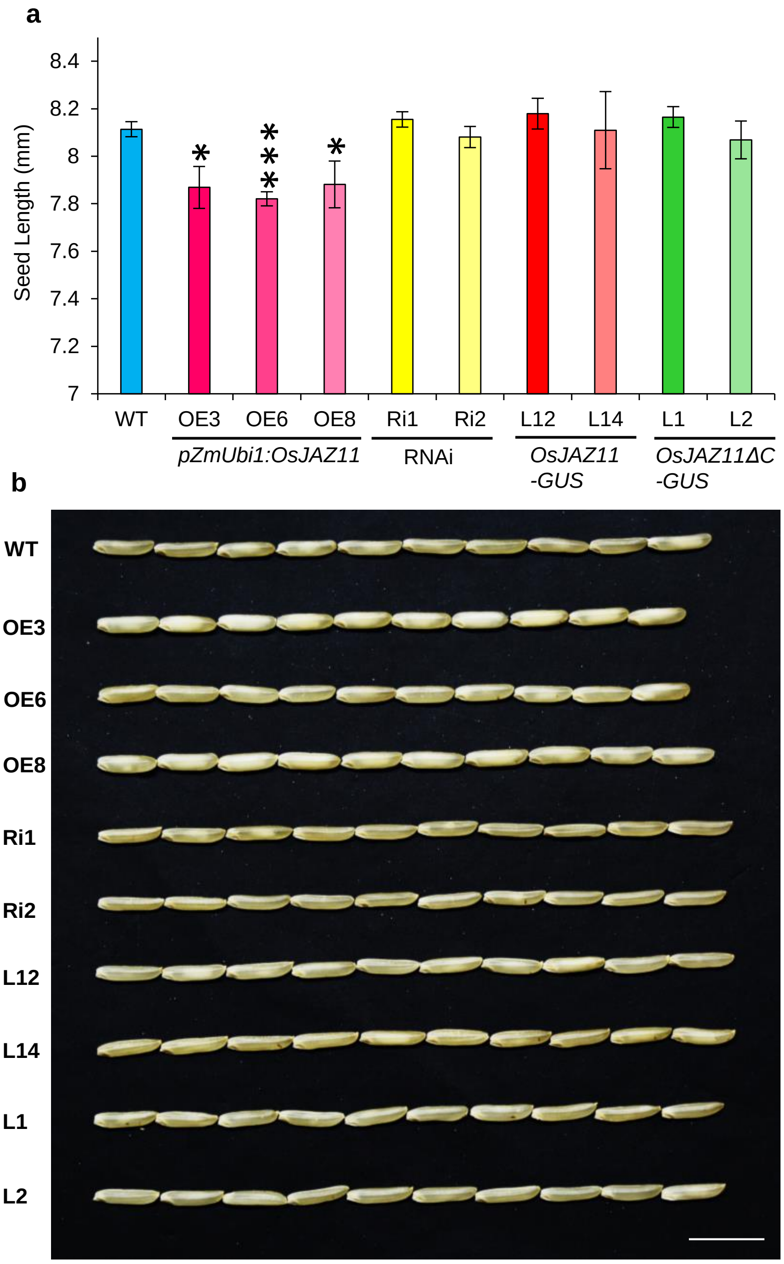

Figure S5. Effect of *OsJAZ11* on seed length. (a) Seed length of WT and *OsJAZ11* transgenics. Each bar represents average of 100 seeds with standard error. Significant differences between WT and transgenics were determined using Student's *t*-test. Asterisks; * and *** represent *p* values, ≤ 0.05 and 0.001, respectively. (b) Seed length phenotype of WT and *OsJAZ11* transgenics. Bar = 1 cm.

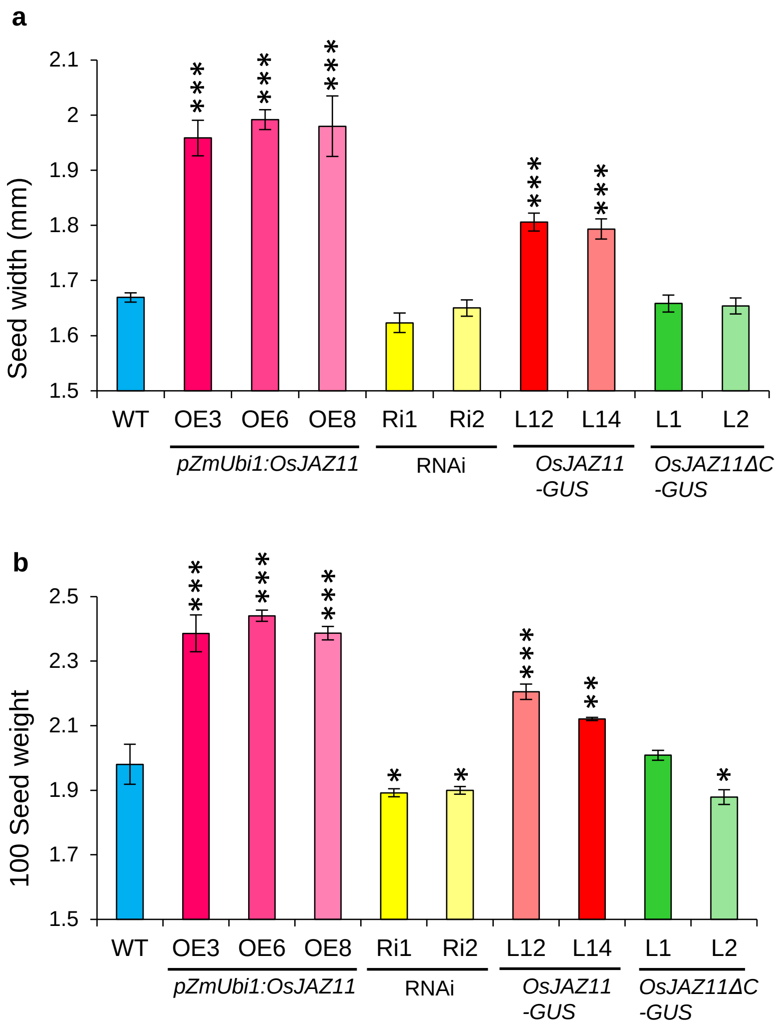

Figure S6. Seed phenotypes of *OsJAZ11* transgenics in T2 generation. (a) Seed width of WT and *OsJAZ11* overexpression lines (*pZmUbi1:OsJAZ11*; OE3/6/8), RNAi lines (Ri1/2), *OsJAZ11-GUS* lines (L12/L14) and *OsJAZ11ΔC-GUS* lines (L1/2). Each bar represents average of 100 seeds. (b) 100 seed weight of WT and *OsJAZ11* transgenics (n =3). Error bar represents standard error. Significant differences between WT and transgenics were determined using Student's *t*-test. Asterisks; *, ** and *** represent *p* values, ≤ 0.05, 0.01 and 0.001, respectively.

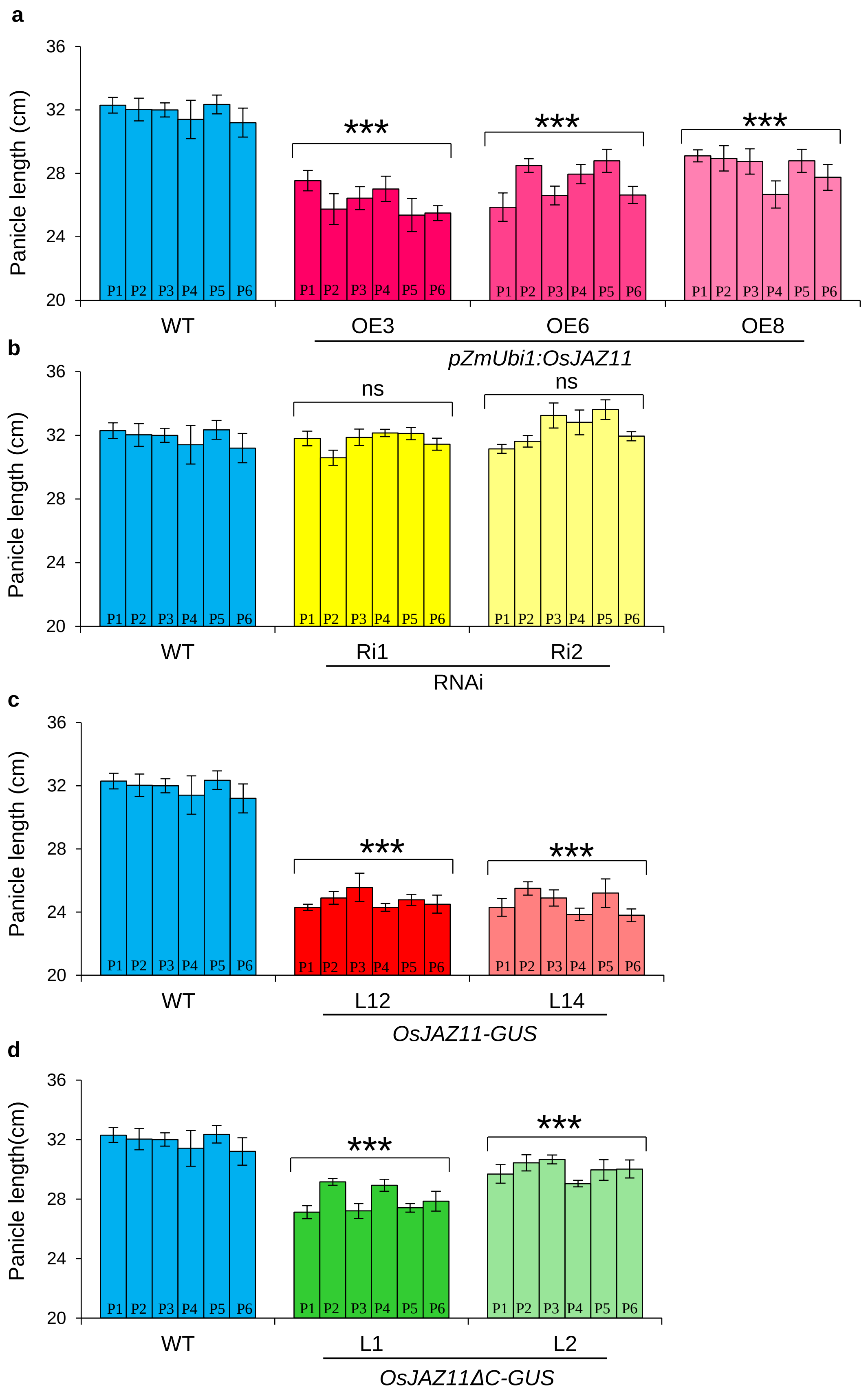

Figure S7. Effect of *OsJAZ11* on panicle length. Panicle length of WT and (a) *OsJAZ11* OE lines, (b) *OsJAZ11* Ri lines, (c) *OsJAZ11-GUS* lines and (d) *OsJAZ11ΔC-GUS* lines. Each bar shows average of 10 panicles from single plant with standard error. For each line data was collected from six different plants (P1 to P6). Same WT controls have been used in all bar graphs (a-d) to compare panicle lengths between WT and transgenics. Significant differences between WT and transgenics were determined using Student's *t*-test. *** represents p value, ≤ 0.001. 'ns' implies 'non-significant' differences between WT and transgenics.

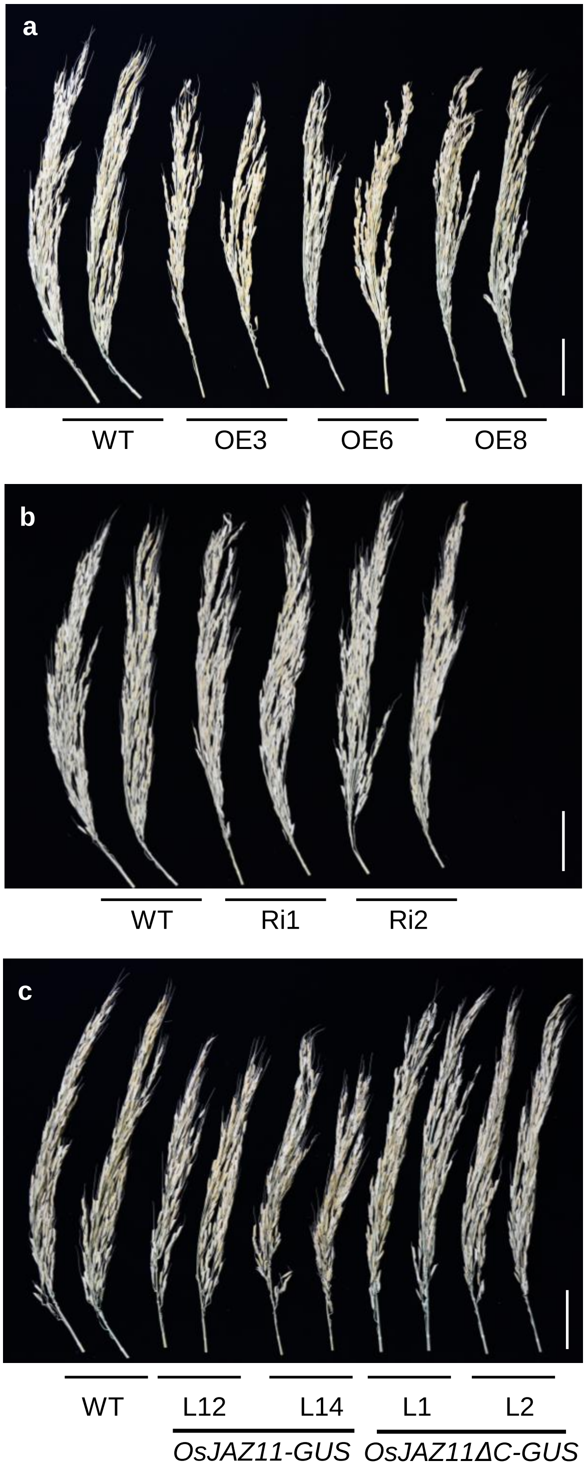

Figure S8. Panicle phenotype of *OsJAZ11* transgenics. Panicle length of WT and (a) *OsJAZ11* OE lines, (b) *OsJAZ11* Ri lines, (c) *OsJAZ11-GUS* and *OsJAZ11ΔC-GUS* lines. Same WT control has been used in (a) and (b) to compare panicle lengths between WT and transgenics. Bar = 5 cm.

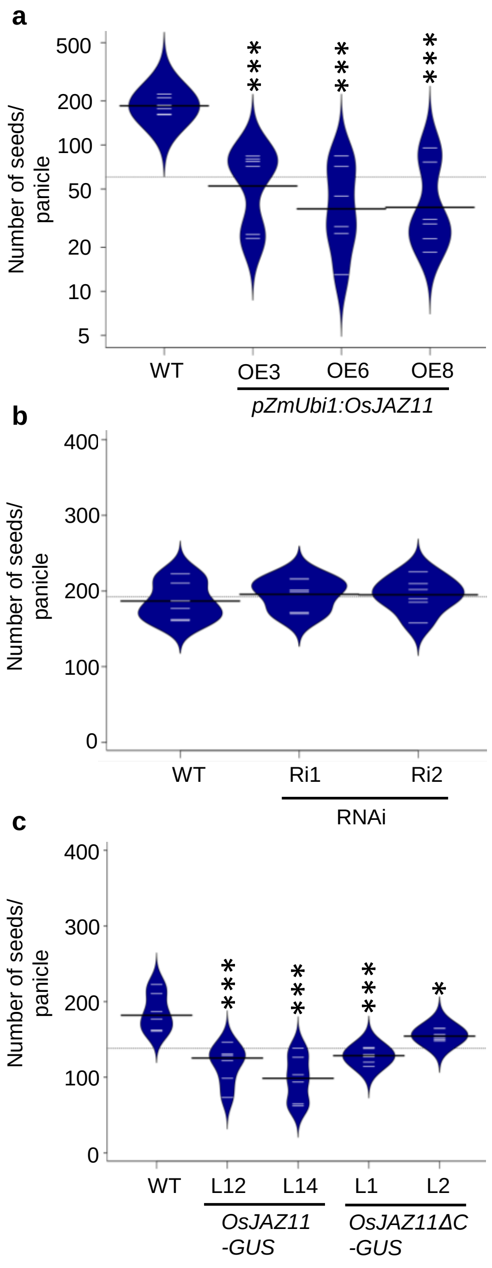

Figure S9. Effect of *OsJAZ11* on seed number. Bean plots showing number of seeds per panicle in WT and (a) *OsJAZ11* OE lines, (b) *OsJAZ11* Ri lines, (c) *OsJAZ11-GUS* and *OsJAZ11ΔC-GUS* lines. Each white line in a bean represents data from single plant. For each transgenic line, data was plotted from six plants. Average of six plants has been represented by black line in each bean. Significant differences between WT and transgenics were determined using Student's *t*-test. Asterisks; * and *** represent *p* values, ≤ 0.05 and 0.001, respectively.

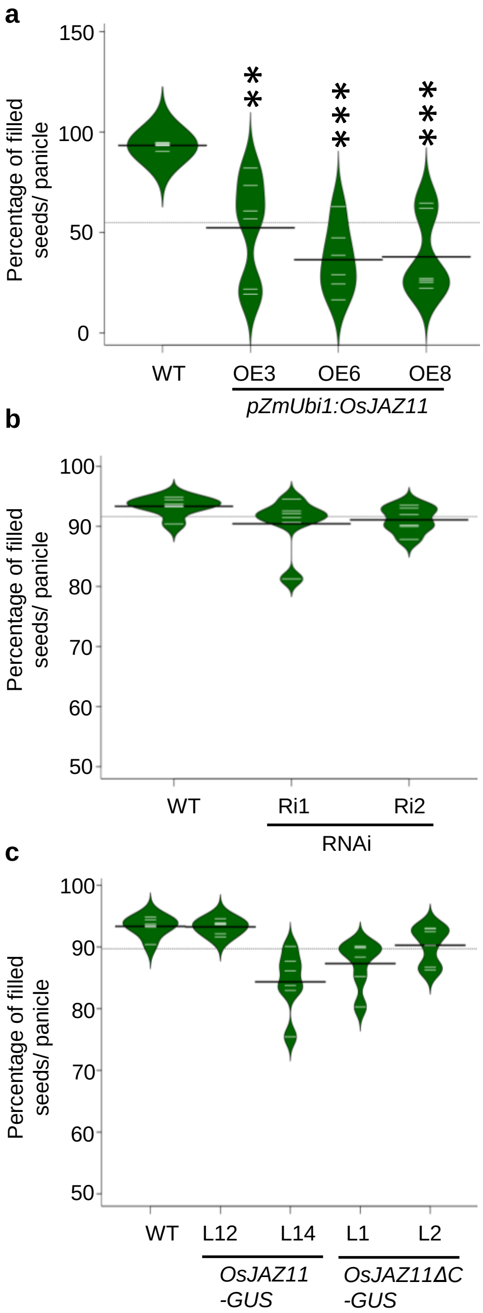

Figure S10. Effect of *OsJAZ11* on percentage of filled seeds per panicle. Bean plots showing percentage of filled seeds per panicle in WT and (a) *OsJAZ11* OE lines, (b) *OsJAZ11* Ri lines, (c) *OsJAZ11-GUS* and *OsJAZ11ΔC-GUS* lines. Each white line in a bean represents data from single plant. For each transgenic line, data was plotted from six plants. Average of six plants has been represented by black line in each bean. Significant differences between WT and transgenics were determined using Student's *t*-test. Asterisks; ** and *** represent *p* values, ≤ 0.01 and 0.001, respectively.

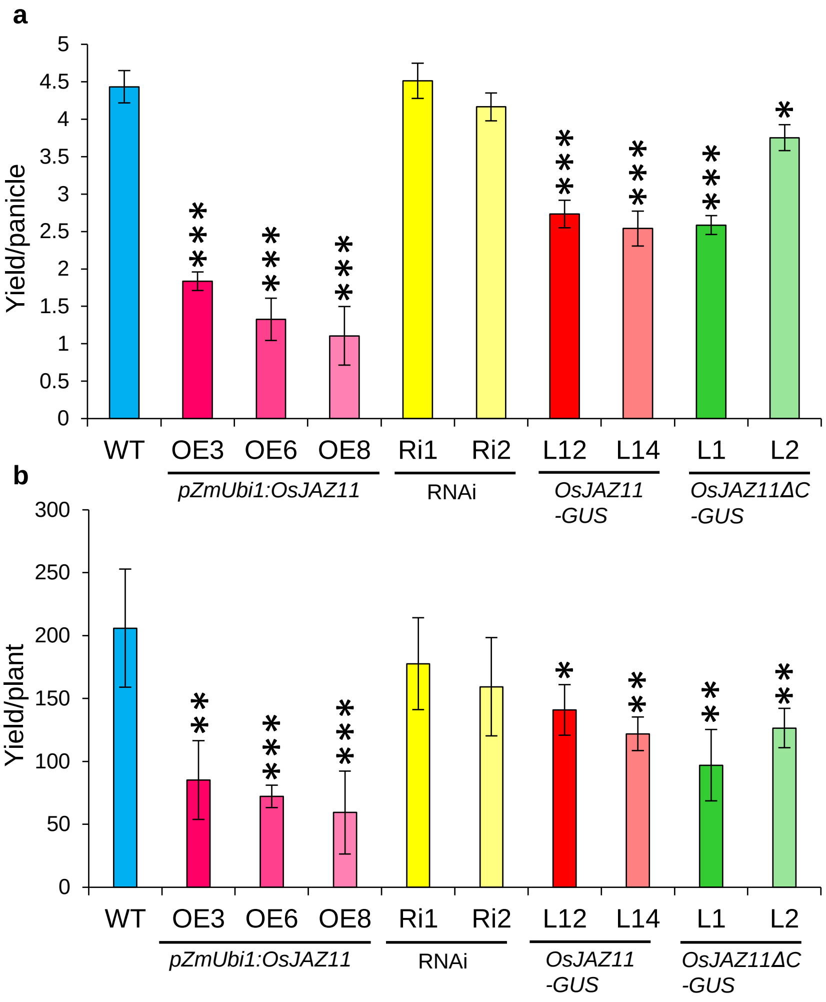

Figure S11. Effect of *OsJAZ11* on yield. (a) Yield per panicle and (b) yield per plant of WT and *OsJAZ11* transgenics (n=6). Error bar represents standard error. Significant differences between WT and transgenics were determined using Student's *t*-test. Asterisks; *, ** and *** represent *p* values, ≤ 0.05, 0.01 and 0.001, respectively.

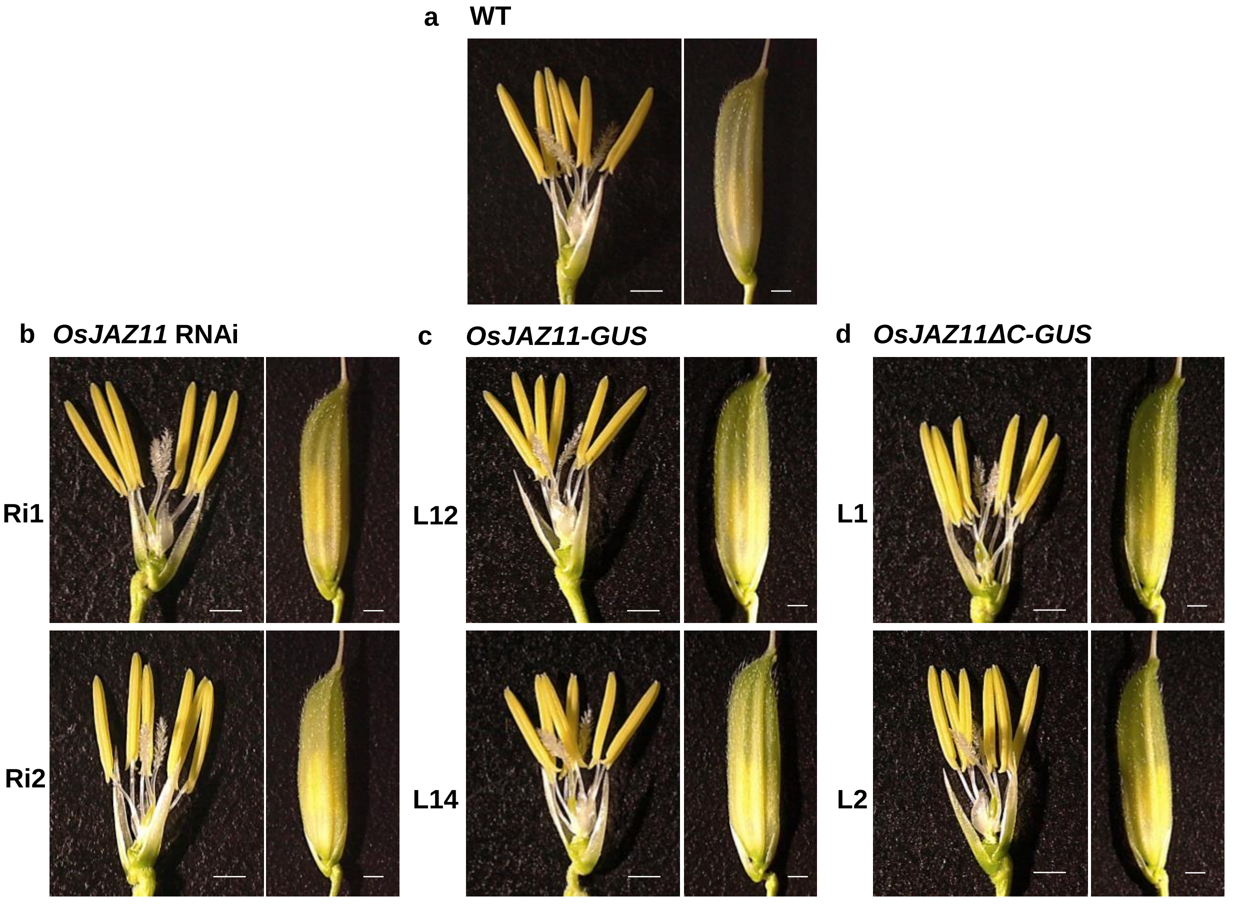

Figure S12. Spikelet phenotype of *OsJAZ11* transgenics. Spikelet morphology of (a) WT, (b) *OsJAZ11* RNAi, (c) *OsJAZ11-GUS* and (d) *OsJAZ11ΔC-GUS* lines without and with lemma/palea. Bar = 1 mm.

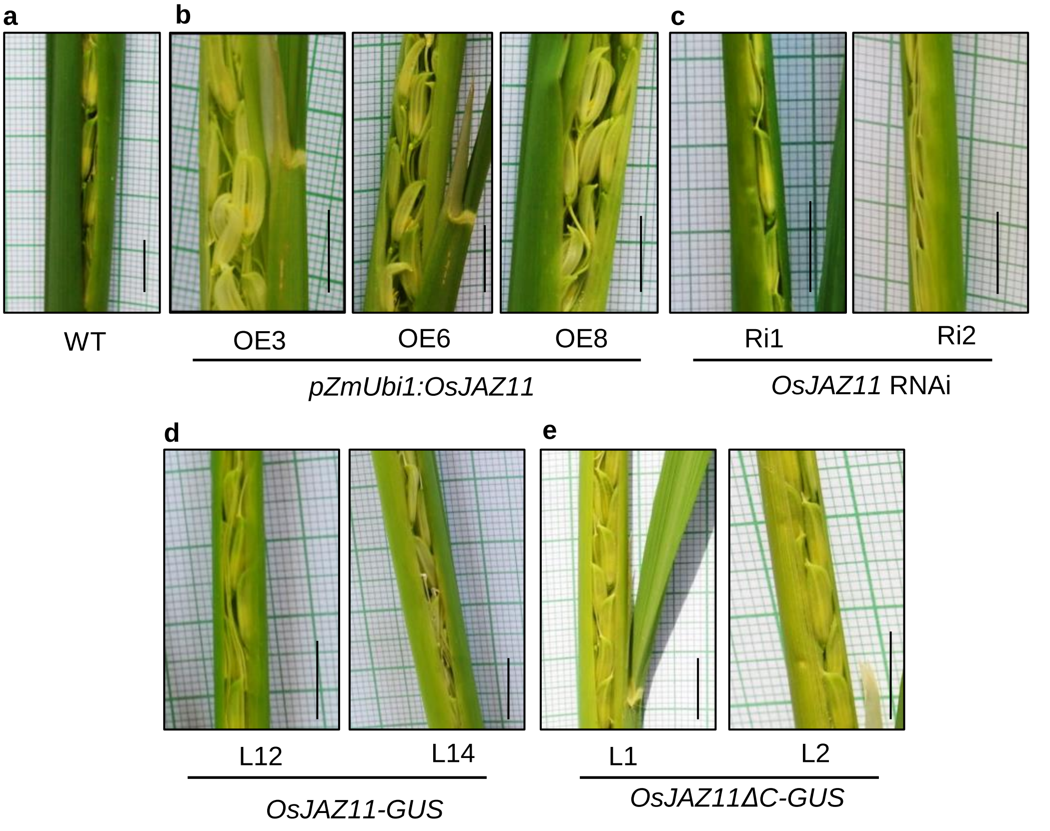

Figure S13. Spikelet phenotype of *OsJAZ11* transgenics before heading. Spikelet morphology of (a) WT, (b) *OsJAZ11* OE lines, (c) *OsJAZ11* RNAi lines, (d) *OsJAZ11-GUS* and (e) *OsJAZ11ΔC-GUS* lines without and with lemma/palea. OE lines of *OsJAZ11* in (b) show abnormal spikelet morphology. Bar = 1 cm.

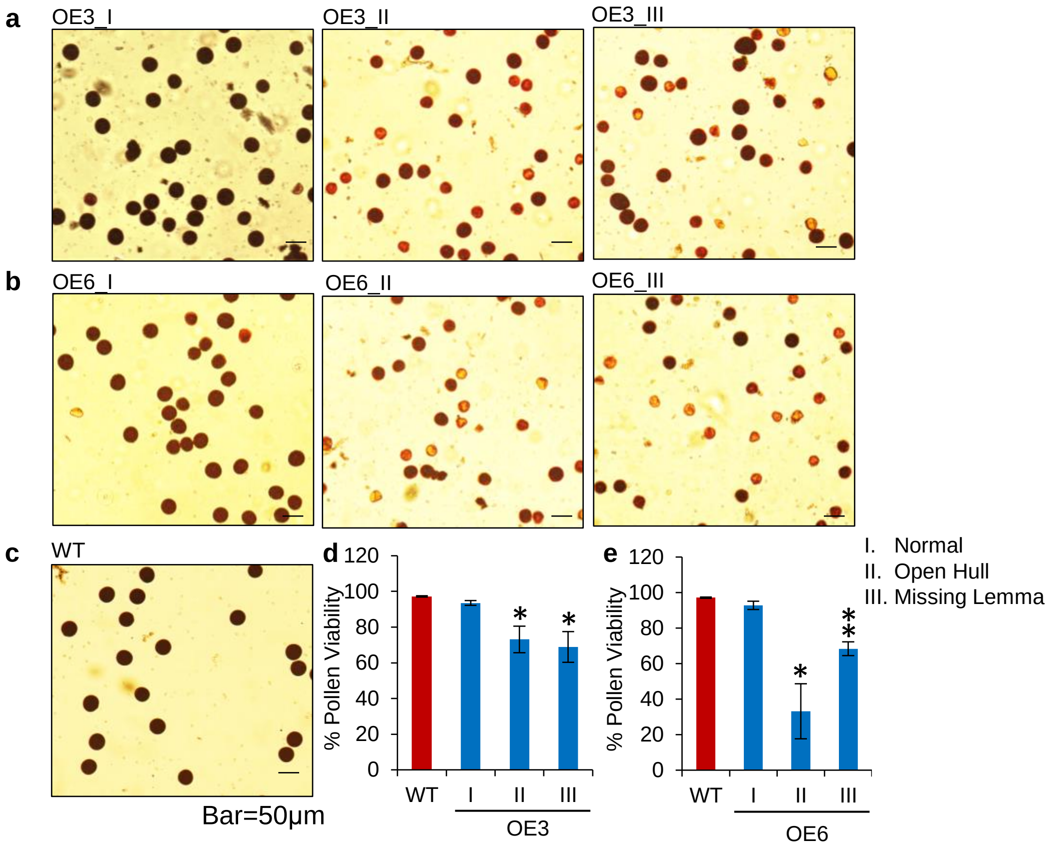

Figure S14. Pollen viability of *OsJAZ11* overexpression lines. I_2-KI staining of pollen grains from *OsJAZ11* OE lines (a) OE3, (b) OE6 and (c) WT. Type I, II and III represents spikelets showing normal morphology, open hull phenotype and missing lemma, respectively. Bar = 50 µm. (d) Percent pollen viability of OE3 and (e) OE6 compared to WT (n=4). Error bar represents standard error. Significant differences between WT and transgenics were determined using Student's *t*-test. Asterisks; * and ** indicate *p* values, ≤ 0.05 and 0.01, respectively.

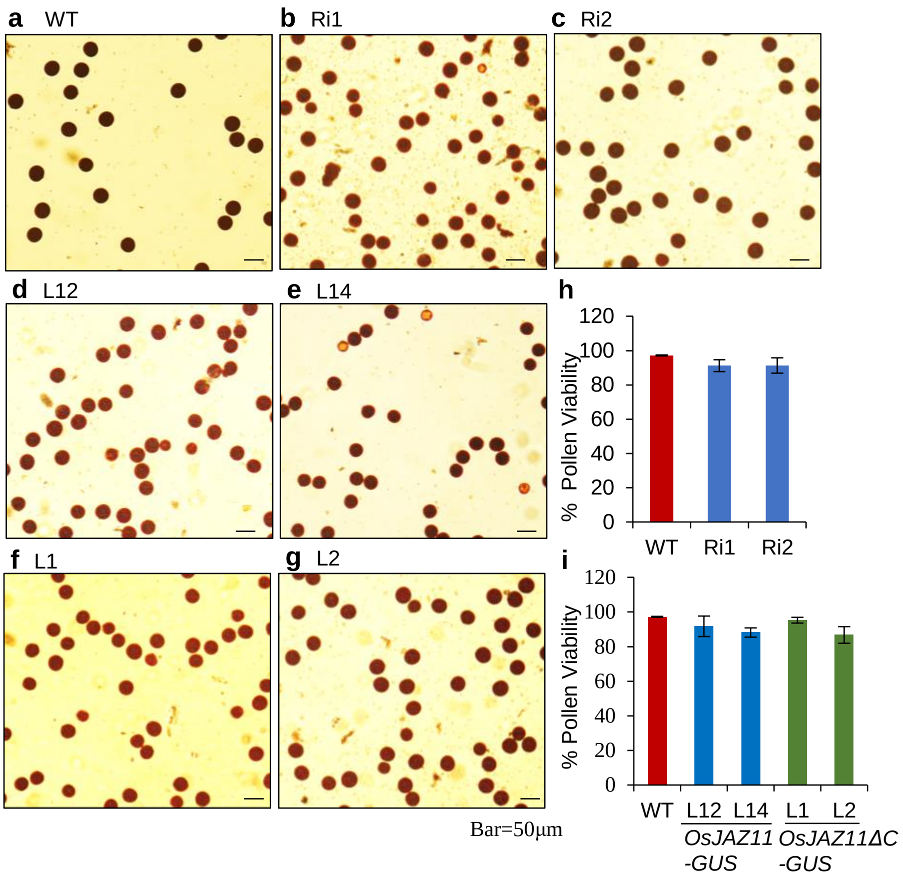

Figure S15. Pollen viability of WT and *OsJAZ11* transgenics. I_2-KI staining of pollen grains from (a) WT, (b-c) *OsJAZ11* Ri lines (Ri1/2), (d-e) *OsJAZ11-GUS* (L12/14) and (f-g) *OsJAZ11ΔC-GUS* (L1/2). Percent pollen viability of (h) *OsJAZ11* RNAi lines and (i) translational reporters compared to WT Bar = 50 µm. Each bar shows average of four replicates with standard error. Significant differences between WT and transgenics were determined by Student's *t*-test.

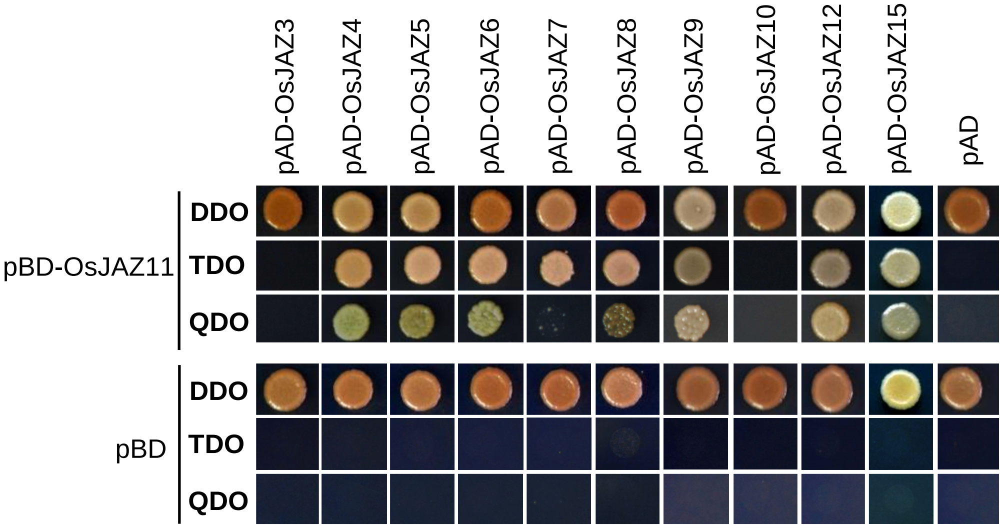

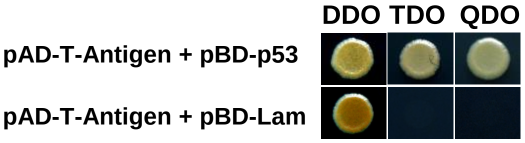

Figure S16. OsJAZ11 heterodimerizes with other JAZ protein. Yeast two-hybrid interaction assays pBD-OsJAZ11 and pAD-OsJAZ3/4/5/6/7/8/9/10/12/15. Yeast cells co-transformed with AD (prey) and BD (bait) plasmids were spotted on DDO medium (SD-Leu/-Trp), TDO medium (SD- Leu/-Trp/-His) and QDO medium (SD- Leu/-Trp/-His/-Ade). pBD and pAD indicates empty BD (pGBKT7) and AD (pGADT7) vectors, respectively. Interaction between pAD-T-Antigen and pBD-p53 was used as positive control whereas interaction between pAD-T-Antigen and pBD-Lam was used as a negative control.

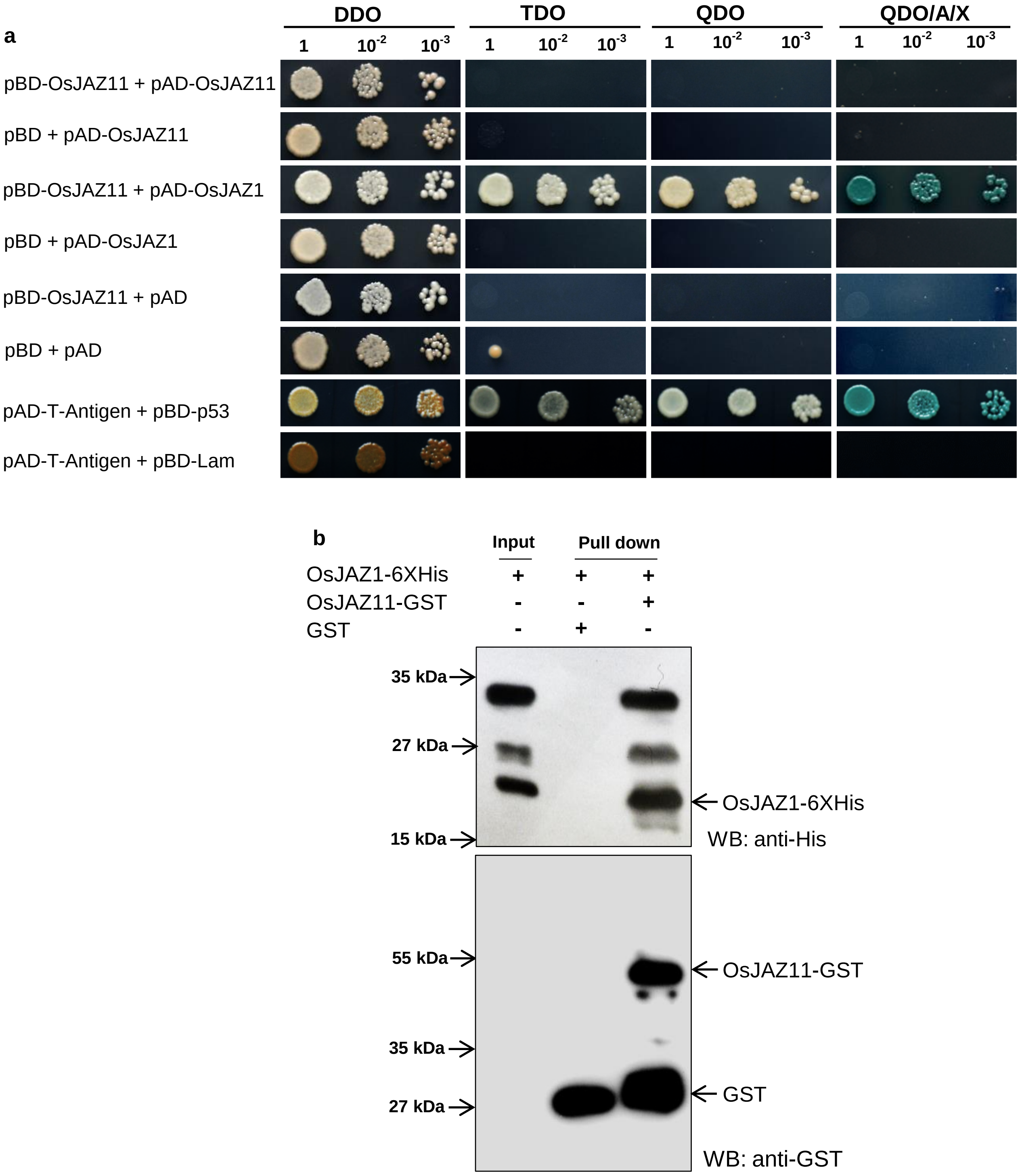

Figure S17. OsJAZ11 interacts with OsJAZ1. (a) Yeast two-hybrid interaction assays between bait plasmid, pBD-OsJAZ11 and prey plasmids, pAD-OsJAZ1 and pAD-OsJAZ11. Serial dilutions of yeast cells co-transformed with AD (prey) and BD (bait) plasmids were spotted on DDO medium (SD-Leu/-Trp), TDO medium (SD- Leu/-Trp/-His), QDO medium (SD- Leu/-Trp/-His/-Ade) and QDO/A/X medium (QDO + Aureobasidin A + X-α-Gal). pBD and pAD indicates empty BD (pGBKT7) and AD (pGADT7) vectors, respectively. Interaction between pAD-T-Antigen and pBD-p53 was used as positive control whereas interaction between pAD-T-Antigen and pBD-Lam was used as a negative control. (b) GST Pull-down assay showing interaction of OsJAZ11-GST and OsJAZ1-6XHis. OsJAZ11-GST and GST (negative control) proteins immobilized on Glutathione−Agarose beads were incubated with OsJAZ1-6XHIS (input protein). Pulled-down protein complexes were probed by immunoblotting using anti-GST and anti-HIS antibodies.

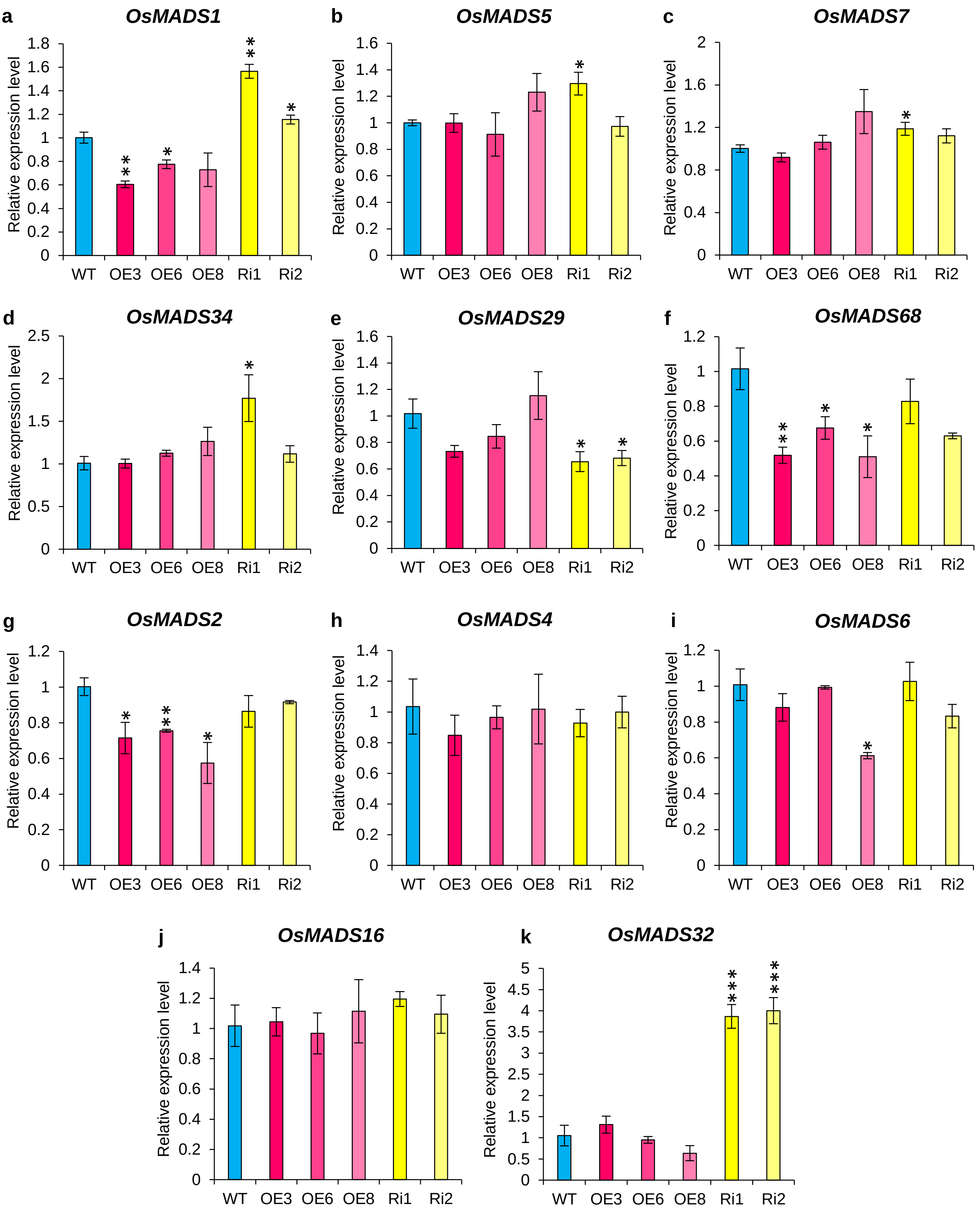

Figure S18. Effect of *OsJAZ11* on expression of MADS transcription factors. Relative expression profile of MADS-domain transcription factors in P4 stage spikelets of WT, *OsJAZ11* OE and RNAi lines (n=3). Error bar shows standard error. Significant differences between WT and transgenics were determined using Student's *t*-test. Asterisks; *, ** and *** represent *p* values, ≤ 0.05, 0.01 and 0.001, respectively.

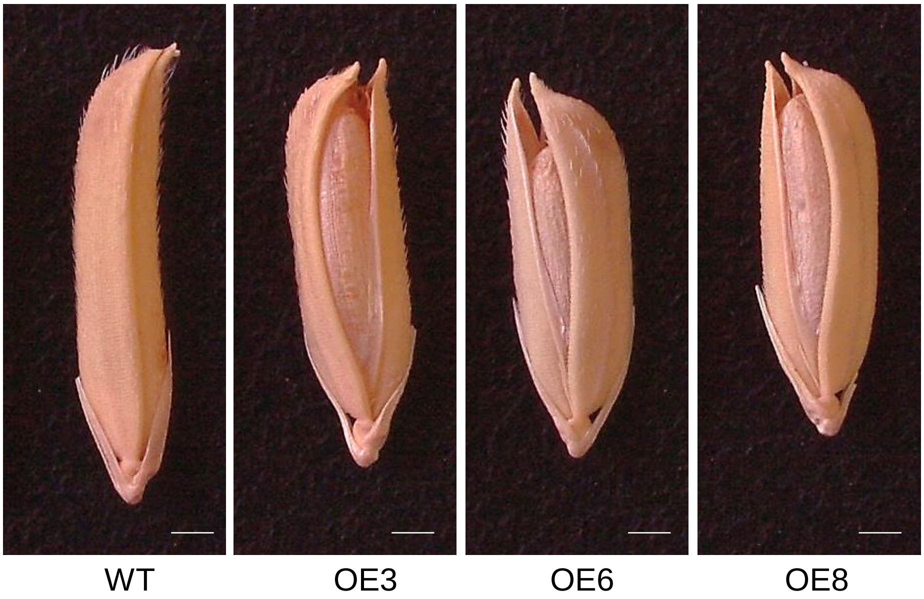

Figure S19. Overexpression of *OsJAZ11* leads to widening of hulls. Mature grain phenotype of WT and *OsJAZ11* OE lines. Bar = 1mm.
