# Supplementary material for: OsJAZ11 regulates spikelet and seed development in rice
Source: Plant Direct. 2022 May 10;6(5):e401. doi: 10.1002/pld3.401 (PMC9090556; doi:10.1002/pld3.401)
Supplement: Supplementary file 2 — Table S1. List of primers used in the study [file PLD3-6-e401-s003.docx]

| **Table S1.** List of primers used in the study | | |
| --- | --- | --- |
|  | F (5'-3') | R (5'-3') |
| *OsJAZ11_pMDC163* | CACCATCAATGCACCTAGGTGC | GTTGTAGCTCTAGCAAAATCAGCTAG |
| *OsJAZ11_pGEX4T1* | TGCTTAGGATCCATGGCCGGTAGTAGCGAGC | GAGTTCGAATTCTCACAGGCTGAGAGTGGGGT |
| *OsJAZ11_qRT_pCAMBIA* | ACGCTGAAAGACACGCAAGA | TGGTCAGCTGCGCCTTCT |
| *OsJAZ11_qRT_pANIC6B/pANIC8B* | AATTCTTTCATGCATGCTGATCTTA | AGGTCCGACAGGCATCAATC |
| *OsMYC2_qRT* | AAATCCATTCCACCCCATACC | CCGTCCAAAGGTTCATCGAT |
| *OsJAZ1_qRT* | CAGCAGGTTGGTGAGCAAAG | TCCATCCCTGATGCTTCCAT |
| *OsJAZ4_qRT* | GAGTGCCAATGACAACAAGTCATC | TGATTCGTCGCGGTTGCT |
| *OsJAZ5_qRT* | CGAGGCAACTAAAGCAAAAGGA | TGAGTGGCTCTTTGGCAAAATT |
| *OsJAZ6_qRT* | TTGATGACTTCCCAGCTGAGAA | GCGCTGTGGAGGAACTCTTG |
| *OsJAZ8_qRT* | GAAGGCTCAACAGCTGACCAT | TTGGTGGACGGGAAGTTCTC |
| *OsJAZ9_qRT* | CGGTCGAGTTGGAAGATGGTT | GGTCAGGCTCGGCGAAAT |
| *OsJAZ12_qRT* | TGCAGCGTTTCCTCCAGAA | CTTGTATGGTTCGCTCGTTGTC |
| *OsJAZ15_qRT* | TCCGTTGGATCGCGTTTTA | CCTATCCGAACCTCCATCGA |
| *OSAOS1_qRT* | CGCCTCGGCATTGCA | AGCGTCGGGAACAGGATCTT |
| *OsAOS2_qRT* | TGCCCATGATCATCGAGGAT | ACGCGTACAGCGCCTTGTA |
| *JMT1_qRT* | TGGGCTTCTTGACGAGATGAA | GCTCGTGCTTGATATGTACATCTTTC |
| *OsLOX1_qRT* | GGGCCTCACGGTGCAA | TCGTGGTGGTCTAGGATGAAAA |
| *OsOPR1_qRT* | TCGCTGCTGGCGGGTAT | TAGCCATCAGCCACCACCTT |
| *OsOPR2_qRT* | CCGGTAGCTCTCGGCTCATA | AGAGAAAGCCTGGGTGCTTGT |
| *OsOPR4_qRT* | TCTTGGCCAATCCGGACTT | ATACTTGTTCAATGGTGCGTTGA |
| *OsOPR5_qRT* | TGATGCGCCATTGAACAAGT | TCCAACAATTGGGTCTTGAGTGT |
| *OsOPR6_qRT* | GCCGCCGGCAAATTC | GCCATTGAACTGCTTCCTGAA |
| *GW2_qRT* | CTGTGGCAACTTTATGCCTGTAA | CGAATGGCTGGCGTTCAC |
| *GW7_qRT* | TCATGCTCATCCTTTTCATCACTT | GTTGATGTATGGCAGCTCTTGTTG |
| *GW8_qRT* | CGATGGTGGGTTGACACAAG | CCGGAGCTGACAGAAGAGAGA |
| *GS5_qRT* | TTCAGCATCTTCACCTGATCGT | TGATTCTCCACCTGAATTGTTCCT |
| *MADS1_qRT* | GGATTTGGGCCCACTAAGC | TGAGGGATACTTCTATCTGGTTCTCA |
| *MADS5_qRT* | GGCCGCCTCTTCGAGTTC | CGGTATCGCTCCAGTGTCTTG |
| *MADS7_qRT* | GGGTTCTTCCATCCACTTGATG | CTGCAGGGTACCCAATCTGAA |
| *MADS34_qRT* | AGCTTGACGAGATCGACGTAGAG | GCAGTTTCCGTTCCATGGA |
| *MADS29_qRT* | GACAACCTTCGTCGCAAGGA | CATGCGGCACAGGAAGCT |
| *MADS68_qRT* | CCCTCTCATCCTGCTGATGTTT | TCGCCAACGCAAATCGT |
| *MADS2_qRT* | CCAGACCAATTCCGGAAAGAT | TCCGCGCTAAGGCTCTTG |
| *MADS4_qRT* | GCTGCTGAGCACTGCCAAT | TGAGGAAATGCAAAGCTAGCAA |
| *MADS6_qRT* | TGCAGGTGCAGAGAACAACTTC | TCGATGGCTGCTTAGCTCAA |
| *MADS16_qRT* | TTATGTATGCGTGGTGTGCAACT | GCAAGCGTGGCAAATAAGTG |
| *MADS32_qRT* | TCCTGGAGAAACAGCTGAGGTT | GCGACGGCACCGTGTACT |
| *OsUBQ5_qRT* | ACCACTTCGACCGCCACTACT | ACGCCTAAGCCTGCTGGTT |
| *OsJAZ11_AD/BD* | CACCATGGCCGGTAGTAGC | CAGGCTGAGAGTGGGGTTC |
| *OsMADS1_AD* | GGCTGAATTCATGGGGAGGGGGAAGGTG | AAGTGGATCCTCATATCCAGCCGGATGGGATGTG |
| *OsMADS2_AD* | AAGTCATATGATGGGGCGCGGGAAGATCGA | AATGGAATTCTTAATTGTTCTCCTGCAGGTTGGGGTGGCT |
| *OsMADS3_AD* | GGCTGAATTCATGATGAACATGATGACCGATCTGAGC | AAGTGGATCCCTAACGTACGTGTGTACGTACGGT |
| *OsMADS4_AD* | GGCTGAATTCATGGGGCGCGGCAAGATCGA | AAGTGGATCCCTACTTCTCCTGCTGGAGGTTGGGGTG |
| *OsMADS5_AD* | GGCTGAATTCATGGGGCGAGGGAAAGTAGA | AAGTGGATCCTCATTGGTTGAGGTGATCCATGTAAGC |
| *OsMADS6_AD* | GGCTGAATTCATGGGGAGGGGAAGAGTTGAG | AAGTGGATCCTCAAAGAACCCATCCCAGCATGAAG |
| *OsMADS8_AD* | GGCTGAATTCATGGGGAGAGGGAGGGTGGA | AAGTGGATCCTCAGGGTAGCCATGTCGGCATGAAG |
| *OsMADS15_AD* | GGCTGAATTCATGGGGCGGGGGAAGGTG | GGGTGGATCCTTAAGCATTGAGGTGGCTCAGCATCCATG |
| *OsMADS17_AD* | GTCCCATATGATGGATCGATCAGAGATGGGGAGG | AATTGGATCCTCAGAGGGGCCATCCCATCA |
| *OsMADS34_AD* | GGCTGAATTCATGGGGCGAGGCAAGGTGG | AATTGGATCCCTAGGCCATCCACTCAGGAGGATAACCG |
| *OsMADS58_AD* | AATGGAATTCATGCACATATACAAAGAGCAGG | AATTGGATCCTCAAATTTCATTTGTTGAACTATGAACTC |
| *OsMADS29_AD* | TCTCATATGATGGGGCGCGGCAAG | ATTGGATCCTTACCACAGCTGCAGG CCGT |
| *OsMADS62_AD* | CACCATGGGGAGGGTGAAGC | GGCGATGTTCGCCGGCG |
| *OsMADS63_AD* | CACCATGGGACGGGTGAAG | ACCAACGTTAACCGGAGCAATG |
| *OsMADS68_AD* | CACCATGGGGAGGGTCAAGCTCAAG | AATCATGAGCTGCCGGTG |
| *OsJAZ1_AD* | CACCATGGATCTGTTGGAGAAGAAGAACA | CTGGGCCTTGCCCTCAGCAGTTAC |
| *OsJAZ3_AD* | CACCATGGAGAGGGATTTTCTTGG | TATCTGTAACTTTGTGCTGGGGGGC |
| *OsJAZ4_AD* | CACCATGGAGAGGGACTTCCTG | GATTTGTAGCTTTGTACTGGGGGACTC |
| *OsJAZ5_AD* | CACCATGTCGACGAGGGCGC | GGACGCCGTGTGCTCCTCTTCCTT |
| *OsJAZ6_AD* | CACCATGGCTTCCGCGAAATCCG | TTGGCTCGATTCCTGCCGCAAGC |
| *OsJAZ7_AD* | CACCATGGCGGCTTCCGCGAG | TTGGCCGCGTTCTATGGGCTTCACG |
| *OsJAZ8_AD* | CACCATGGCCGGCCGTGC | TATCTCCTGCTTTATTGTCATCTCTTGGCCAAACC |
| *OsJAZ9_AD* | CACCATGGCGTCGACGGATCC | GCGCGAGTGCATGTGTCCAAGG |
| *OsJAZ10_AD* | CACCATGGCGATGGAGGGGAAGAG | CAGCGCGATGGTGAGGCTGTCA |
| *OsJAZ12_AD* | CACCATGGCCGCCGCCGGC | GAGCCCGAGCCATGTCGCCGGCTC |
| *OsJAZ15_AD* | CACCATGGACGCCGTCGGCG | CTTTTGCTTCCTCTTTTGGAGGAATCGCTGCAG |
| *OsMADS29_pET28a* | TCTCATATGATGGGGCGCGGCAAG | ATTGGATCCTTACCACAGCTGCAGG CCGT |
| *OsMADS68_pET28a* | ATTAGAATTCATGGGGAGGGTCAAGC | ATATGTCGACTCAAATCATGAGCTGCCG |
| *OsJAZ1_pET28a* | AGTCGAATTCATGGATCTGTTGGAGAAGAA | ATATAGTCGACTTACTGGGCCTTGCCCTC |
