# Supplementary material for: OsJAZ11 regulates spikelet and seed development in rice
Source: Plant Direct. 2022 May 10;6(5):e401. doi: 10.1002/pld3.401 (PMC9090556; doi:10.1002/pld3.401)
Supplement: Supplementary file 3 — Table S2. Proportion of open hull spikelets among total defective spikelets in OsJAZ11 OE lines [file PLD3-6-e401-s002.docx]

| **Table S2.** Proportion of open hull spikelets among total defective spikelets in *OsJAZ11* OE lines | | | |
| --- | --- | --- | --- |
|  | **Defective Spikelets (%)** | **Open hull (%)** | **Number of Panicles and spikelets analysed** |
| **WT** | nil | nil | 5 Panicles (623 spikelets) |
| **OE3** | 59.13 ± 3.76 | 56.34 ± 3.78 | 7 Panicles (629 spikelets) |
| **OE6** | 36.36 ± 3.85 | 31.15 ± 5.98 | 8 Panicles (833 spikelets) |
| **OE8** | 49.03 ± 3.92 | 42.12 ± 4.54 | 14 Panicles (1409 spikelets) |
