# Supplementary material for: OsJAZ11 regulates spikelet and seed development in rice
Source: Plant Direct. 2022 May 10;6(5):e401. doi: 10.1002/pld3.401 (PMC9090556; doi:10.1002/pld3.401)
Supplement: Supplementary file 4 — Table S3. Number of stamens, carpels and extra glume‐like structures observed in OsJAZ11 OE lines [file PLD3-6-e401-s004.docx]

| **Table S3.** Number of stamens, carpels and extra glume-like structures observed in *OsJAZ11* OE lines | | | | |
| --- | --- | --- | --- | --- |
|  | **Stamens** | **Carpels** | **Extra glumes** | **Total spikelets analysed** |
| **WT** | 6 | 1 | 0 | 23 |
| **OE3** | 3-10 | 1-2 | 0-4 | 33 |
| **OE6** | 3-8 | 1-2 | 0-4 | 32 |
| **OE8** | 5-13 | 1-2 | 0-4 | 34 |
